# Structural differences in adolescent brains can predict alcohol misuse

**DOI:** 10.7554/eLife.77545

**Published:** 2022-05-26

**Authors:** Roshan Prakash Rane, Evert Ferdinand de Man, JiHoon Kim, Kai Görgen, Mira Tschorn, Michael A Rapp, Tobias Banaschewski, Arun LW Bokde, Sylvane Desrivieres, Herta Flor, Antoine Grigis, Hugh Garavan, Penny A Gowland, Rüdiger Brühl, Jean-Luc Martinot, Marie-Laure Paillere Martinot, Eric Artiges, Frauke Nees, Dimitri Papadopoulos Orfanos, Herve Lemaitre, Tomas Paus, Luise Poustka, Juliane Fröhner, Lauren Robinson, Michael N Smolka, Jeanne Winterer, Robert Whelan, Gunter Schumann, Henrik Walter, Andreas Heinz, Kerstin Ritter, IMAGEN consortium

**Affiliations:** 1 Charité – Universitätsmedizin Berlin (corporate member of Freie Universiät at Berlin, Humboldt-Universiät at zu Berlin, and Berlin Institute of Health), Department of Psychiatry and Psychotherapy, Bernstein Center for Computational Neuroscience Berlin Germany; 2 Faculty IV – Electrical Engineering and Computer Science, Technische Universität Berlin Berlin Germany; 3 Department of Education and Psychology, Freie Universität Berlin Berlin Germany; 4 Science of Intelligence, Research Cluster of Excellence Berlin Germany; 5 Social and Preventive Medicine, Department of Sports and Health Sciences, Intra-faculty unit “Cognitive Sciences”, Faculty of Human Science, and Faculty of Health Sciences Brandenburg, Research Area Services Research and e-Health, University of Potsdam Potsdam Germany; 6 Department of Child and Adolescent Psychiatry and Psychotherapy, Central Institute of Mental Health, Medical Faculty Mannheim, Heidelberg University Mannheim Germany; 7 Discipline of Psychiatry, School of Medicine and Trinity College Institute of Neuroscience, Trinity College Dublin Dublin Ireland; 8 Centre for Population Neuroscience and Precision Medicine (PONS), Institute of Psychiatry, Psychology Neuroscience SGDP Centre, King’s College London London United Kingdom; 9 Institute of Cognitive and Clinical Neuroscience, Central Institute of Mental Health, Medical Faculty Mannheim, Heidelberg University Heidelberg Germany; 10 Department of Psychology, School of Social Sciences, University of Mannheim Mannheim Germany; 11 NeuroSpin, CEA, Université Paris-Saclay Paris France; 12 Departments of Psychiatry and Psychology, University of Vermont Burlington United States; 13 Sir Peter Mansfield Imaging Centre School of Physics and Astronomy, University of Nottingham Nottingham United Kingdom; 14 Physikalisch-Technische Bundesanstalt Berlin Germany; 15 Institut National de la Santé et de la Recherche Médicale, INSERM U A10 ”Trajectoires développementales en psychiatrie” Universite Paris-Saclay, Ecole Normale Supérieure Paris-Saclay, CNRS, Centre Borelli Gif-sur-Yvette France; 16 AP-HP Sorbonne Université, Department of Child and Adolescent Psychiatry, Pitié-Salpêtrière Hospital Paris France; 17 Psychiatry Department, EPS Barthélémy Durand Etampes France; 18 PONS Research Group, Dept of Psychiatry and Psychotherapy, Campus Charite Mitte, Humboldt University Berlin Germany; 19 Institut des Maladies Neurodégénératives, UMR 5293, CNRS, CEA, University of Bordeaux Bordeaux France; 20 Department of Psychiatry, Faculty of Medicine and Centre Hospitalier Universitaire Sainte-Justine, University of Montreal Montreal Canada; 21 Departments of Psychiatry and Psychology, University of Toronto Toronto Canada; 22 Department of Child and Adolescent Psychiatry and Psychotherapy, University Medical Centre Göttingen Göttingen Germany; 23 Department of Psychiatry and Neuroimaging Center, Technische Universität Dresden Dresden Germany; 24 Department of Psychological Medicine, Section for Eating Disorders, Institute of Psychiatry, Psychology and Neuroscience, King’s College London London United Kingdom; 25 Department of Education and Psychology, Freie Universität Berlin Berlin Germany; 26 School of Psychology and Global Brain Health Institute, Trinity College Dublin Dublin Ireland; University of Oxford United Kingdom; National Institute of Mental Health, National Institutes of Health United States

**Keywords:** adolescence alcohol misuse, machine learning, data science for psychiatry, alcohol use disorder, magnetic resonance imaging, confound control, Psychiatric Research, Multivariate Analysis

## Abstract

Alcohol misuse during adolescence (AAM) has been associated with disruptive development of adolescent brains. In this longitudinal machine learning (ML) study, we could predict AAM significantly from brain structure (T1-weighted imaging and DTI) with accuracies of 73 -78% in the IMAGEN dataset (n∼1182). Our results not only show that structural differences in brain can predict AAM, but also suggests that such differences might precede AAM behavior in the data. We predicted 10 phenotypes of AAM at age 22 using brain MRI features at ages 14, 19, and 22. Binge drinking was found to be the most predictable phenotype. The most informative brain features were located in the ventricular CSF, and in white matter tracts of the corpus callosum, internal capsule, and brain stem. In the cortex, they were spread across the occipital, frontal, and temporal lobes and in the cingulate cortex. We also experimented with four different ML models and several confound control techniques. Support Vector Machine (SVM) with rbf kernel and Gradient Boosting consistently performed better than the linear models, linear SVM and Logistic Regression. Our study also demonstrates how the choice of the predicted phenotype, ML model, and confound correction technique are all crucial decisions in an explorative ML study analyzing psychiatric disorders with small effect sizes such as AAM.

## Introduction

Many adolescents participate in risky and excessive alcohol consumption behaviors (Crews et al., 2007), especially in European and North American countries. Several studies have identified that such early and risky exposure to alcohol is a potential risk factor that can lead to the development of Alcohol Use Disorder (AUD) later in life (DeWit et al., 2000; Grant et al., 2006; Nixon and McClain, 2010). During adolescence and early adulthood (age 10–24), the human brain undergoes maturation characterized by an increase in white matter (WM) (Lebel and Beaulieu, 2011) and an initial thickening and later thinning of grey matter (GM) regions (Giedd, 2004). Researchers have suggested that excessive alcohol use during this period might disrupt normal brain maturation, causing lifelong effects (Crews et al., 2007; Monti et al., 2005; Chambers et al., 2003). Therefore, understanding how alcohol misuse during adolescence is related to the development of Alcohol Use Disorder (AUD) later in life is crucial to understanding alcohol addiction. Furthermore, uncovering how adolescent alcohol misuse (AAM) is associated with their brain at different stages of adolescent brain development can help to implement a more informed public health policy surrounding alcohol use during this age. Previous studies: Several studies in the last two decades have attempted to uncover how adolescent alcohol misuse (AAM) and their structural brain are related. These are summarised in Table 1. Earlier studies collected data with small sample size of 30–100 subjects and compared specific brain regions (such as the hippocampus or the pre-frontal cortex (pFC)) between adolescent alcohol misusers (AAMs) and mild users or non-users (controls). They used structural features such as regional volume (De Bellis et al., 2000; Nagel et al., 2005; De Bellis et al., 2005), cortical thickness (Squeglia et al., 2012), or white matter tract volumes (McQueeny et al., 2009; Jones and Nagel, 2019). These studies found differences between the groups in regions such as the hippocampus (De Bellis et al., 2000; Nagel et al., 2005), cerebellum (De Bellis et al., 2005), and the frontal cortex (De Bellis et al., 2005). However, these findings are not always consistent across studies (Jones et al., 2018). This inconsistency is also evident from the findings in the last column of Table 1. Another group of studies investigated into whether AAM disrupts the natural developmental trajectory of adolescent brains (Jacobus et al., 2013; Luciana et al., 2013; Pfefferbaum et al., 2018; Jones and Nagel, 2019; Sullivan et al., 2020; Robert et al., 2020). These studies reported that the brains of AAMs showed accelerated GM decline (Luciana et al., 2013; Pfefferbaum et al., 2018; Sullivan et al., 2020) and attenuated WM growth (Luciana et al., 2013; Sullivan et al., 2020) compared to controls. However, brain regions reported were not consistent between these studies either and do not tell a coherent story (Jones et al., 2018) (see Table 1). These differences in findings could be potentially due to the following reasons:

1. Heterogeneous disease with a weak effect size: Alcohol misuse has a heterogeneous expression in the brain (Zahr and Pfefferbaum, 2017). This heterogeneity might be driven by alcohol misuse affecting diverse brain regions in different sub-populations depending on demographic, environmental, or genetic differences (Grant et al., 2015). Furthermore, the effect of alcohol misuse on adolescent brain structure can be weak and hard to detect (especially with the mass-univariate methods used in previous studies). The possibility of several disease subtypes exasperated by the small signal-to-noise ratio can generate incoherent findings regarding which brain regions are affected by alcohol.
2. Higher risk of false-positives: Most previous studies have small sample size that are prone to generate inflated effect size (Button et al., 2013). Furthermore, these studies employ mass-univariate analysis techniques that are vulnerable to *multiple comparisons problem* (Lindquist and Mejia, 2015) and can produce false-positives if ignored. These factors coupled with the possibility of publication bias to produce positive results (Ioannidis, 2005) can have a high likelihood of generating false-positive findings (Scheel et al., 2021).
3. Several metrics to measure alcohol misuse: There is no consensus on what is the best phenotype to measure AAM. Many studies use binge drinking or heavy episodic drinking as a measure of AAM (Squeglia et al., 2012; Whelan et al., 2014; Jones and Nagel, 2019; Robert et al., 2020), while few others use a combination of binge drinking, frequency of alcohol use, amount of alcohol consumed and the age of onset of alcohol misuse (Squeglia et al., 2015; Pfefferbaum et al., 2018; Kühn et al., 2019; Seo et al., 2019; Sullivan et al., 2020). These differences in analyses could potentially produce different findings.

**Table 1.** Literature review of studies that look into structural brain differences between adolescent alcohol misusers (AAMs) and control subjects. The studies are sorted by the year of publication. For each study, the sample size ‘n’, the main analysis technique, and the main structural differences found in AAMs are listed.

Study (year)	n	Analysis / method	Sructural differences in AAMs
De Bellis et al., 2000	36	Statistically compare (univariate)regional brain volumes between groups	Lower hippocampal volume.
Nagel et al., 2005	31	Statistically compare (univariate)regional brain volumes between groups	Lower volume only in left hippocampus aftercontrolling for other psychiatric comorbidities.
De Bellis et al., 2005	42	Statistically compare (univariate)regional brain volumes between groups	Lower pFC, cerebellum volumes in malesbut AAMs had comorbid mental disorders.
McQueeny et al., 2009	28	Mass-univariate analysis ofskeletonized FA voxels (DTI)	Binge drinkers had lower FA in18 white matter areas.
Squeglia et al., 2012	59	Statistically compare (univariate) regional brain volumes between groups	No effect of binge drinking oncortical thickness and sex-specificdifferences among AAMs in left frontal cortex.
Jacobus et al., 2013	54	Mass-univariate analysis of skeletonized FA voxels (DTI)	No effect in AAM-only group, but lowerFA in AAM and comorbid marijuana users.
Luciana et al., 2013	55	Longitudinal mass-univariate analysis of cortical thickness, white matter extent, DTI-extracted FA and MD	Accelerated GM thinning in mid frontal gyrus, attenuated WM growth with lower FAin left caudate, thalamus.
Whelan et al., 2014	692	Exploratory analysis using ML to find best predictors of AAM amongdemographic, psychosocial, genetic, cortical volumes, and fMRI variables	Current AAMs have lower GMVs in parts of frontal lobe and higher GMV in right putamen. Future AAMs have lower GMV in right parahippocampal gyrus and higher in left postcentral gyrus.
Squeglia et al., 2015	137	Exploratory analysis using ML to find best predictors of AAM among demographic, neuropsychological, cortical thickness, and fMRI variables	Future AAM have thinner GM inprecuneus, lateral occipital, ACC, PCC, and frontal and temporal cortex.
Pfefferbaum et al., 2018	483	Longitudinal mass-univariate analysisof GMV development	Accelerated GMV reduction in frontal brain regions.
Jones and Nagel, 2019	113	Modeling the WM microstructure development (DTI) for each voxel	Altered frontostriatal WM microstructureis predictive of future AAM.
Kühn et al., 2019	≈1500	Growth curve modeling ofGM volumes	Higher GMV in caudate nucleus and left cerebellum predicts future AAMs
Seo et al., 2019	≈1000	ML analysis of cue-related brain region followed by mass-univariate analysis for identifying region importance	Current AAMs show reduced GMV inmedial-pFC, oFC, thalamus, bilateral ACC,left amygdala and anterior insular.
Sullivan et al., 2020	548	Longitudinal mass-univariate (GLM)analysis of cerebellar region volumes	Cerebellum: accelerated GM decline in 2 sub-regions and accelerated expansion ofWM in one sub-region and CSF.
Robert et al., 2020	726	Mass-univariate analyses of voxels, followed by analysis of the direction of causality using causal bayesian networks	Accelerated GM atrophy in parts of the temporal cortex and left prefrontal cortex.
Filippi et al., 2021	671	ML analysis for predictors ofresilence towards polysubstance use	Adolescents resilient to PSU show larger GMV in the bilateral cingulate gyrus.

Acronyms::: GM:grey matter; WM:white matter; CSF-cerebrospinal fluid; GMV:grey matter volume; pFC:prefrontal Cortex; oFC:orbitofrontal cortex; ACC:anterior cingulate cortex; PCC:posterior cingulate cortex; GLM:generalized linear models; ML:machine learning; DTI:Diffusion Tensor Imaging; FA:Fractional Anisotropy; MD:mean diffusivity.

Multivariate exploratory analysis: Over the last years, data collection drives such as IMAGEN (Mascarell Maričić et al., 2020), NCANDA (Brown et al., 2015), and UK Biobank (Sudlow et al., 2015) made available large-sample multi-site data with n>1000 that are representative of the general population. This enabled researchers to use multivariate, data-driven, and exploratory analysis tools such as machine learning (ML) to detect effects of alcohol misuse on multiple brain regions (Whelan et al., 2014; Squeglia et al., 2017; Seo et al., 2019; Filippi et al., 2021; Jia et al., 2021; Yip et al., 2022). Such whole-brain multivariate methods are preferable over the previous mass-univariate methods as they have a higher sensitivity to detect true positives (Hebart and Baker, 2018). Furthermore, ML can be easily used for clinical applications such as computer-aided diagnosis, predicting future development of AUD, and future relapse of patients into AUD (Shiraishi et al., 2011).

**Figure 1.**
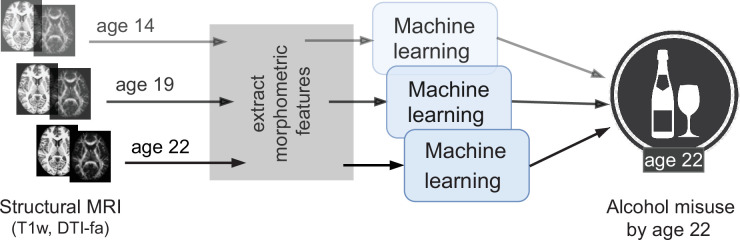
An overview of the analysis performed. Morphometric features extracted from structural brain imaging are used to predict Adolescent Alcohol Misuse (AAM) developed by the age of 22 using machine learning. To understand the causal relationship between AAM and the brain, three separate analyses are performed by using imaging data collected at three stages of adolescence: age 14, age 19, and age 22.

Due to these advantages, several exploratory studies using ML have been attempted in AUD research (Whelan et al., 2014; Seo et al., 2019; Squeglia et al., 2017). We further extend this line of work by analyzing the newly available longitudinal data from IMAGEN (n∼1182 at 4 time points of adolescence) (Mascarell Maričić et al., 2020) by designing a robust and reliable ML pipeline. The goal of this study is to explore the relationship between adolescent brain and AAM using ML and discover any brain features that can be associated with AAM. As shown in Figure 1, we predict AAM at age 22 using brain morphometrics derived from structural imaging captured at three stages of adolescence – ages 14, 19, and 22. The structural features of different brain regions are extracted from two modalities of structural MRI, that is, T1-weighted imaging (T1w) and Diffusion Tensor Imaging (DTI). The most informative structural features for the ML model prediction are discovered using SHAP (Lundberg and Lee, 2017; Lundberg et al., 2020) to reveal the most distinct structural brain differences between AAMs and controls. Furthermore, we use multiple phenotypes of alcohol misuse such as the frequency of alcohol consumption, amount of consumption, onset of misuse, binge drinking, the AUDIT score, and other combinations, and systematically compare them. We also compare four different ML models, and multiple methods of controlling for confounds in ML and derive important methodological insights which are beneficial for reliably applying ML to psychiatric disorders such as AUD. To promote reproducibility and open science, the entire codebase used in this study, including the initial data analysis performed on the IMAGEN dataset are made available at https://github.com/RoshanRane/ML_for_IMAGEN(Rane and Kim, 2022; copy archived at swh:1:rev:6c493672ed700ded73c2b77e8976a5551921e634).

## Results

The results are reported in the following four subsections: In subsection 1, different confound-control techniques are compared and the most suitable technique for this study is determined. Subsection 2 shows the results of the ML exploration performed with ten AAM labels, four ML models, and using imaging data from three time points of adolescence. This stage helps to determine the best phenotype of AAM and the best ML model. Subsection 3 reports the final results on the independent data ${}_{h\,o\,l\,d\,o\,u\,t}$ for all three time point analyses and subsection 4 shows the most informative features found in each of the analyses. Subsection 5 reports the result from the additional *leave-one-site-out* experiment.

### Confound correction techniques

The sex $c_{s\,e\,x}$ and recruitment site $c_{s\,i\,t\,e}$ of subjects confound this study (refer to subsection 5.1 in ‘Materials and methods’) and their influence on the study needs to be controlled. We test three confound correction techniques on data ${}_{e\,x\,p\,l\,o\,r\,e}$ – (a) confound regression (b) counterbalancing with undersampling and (c) counterbalancing with oversampling. To verify if these methods work as expected, the *same analysis approach* from Görgen et al., 2018 and the approach by Snoek et al., 2019 are employed. For the two confounds $c_{s\,e\,x}$ and $c_{s\,i\,t\,e}$, this requires us to test five input-output combinations (X→y, $X\to c_{s\,e\,x}$, $X\to c_{s\,i\,t\,e}$, $c_{s\,e\,x}\to y$ and $c_{s\,i\,t\,e}\to y$) for a given X→y analysis.

**Figure 2.**
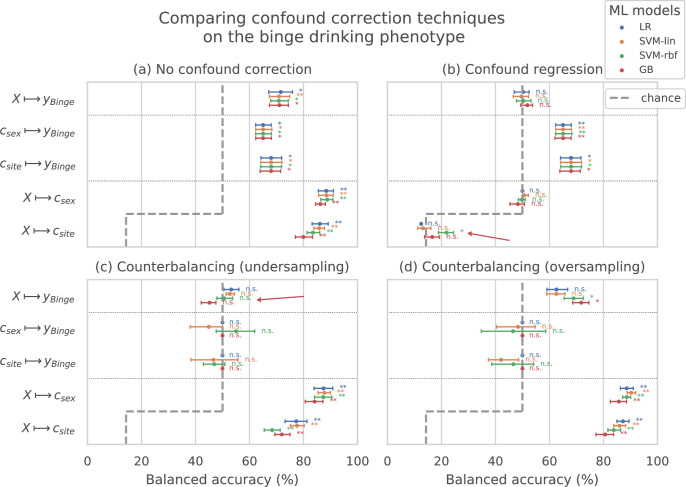
Comparing confound correction techniques. Five input-output settings are compared within each confound correction technique: X→y, $X\to c_{s\,e\,x}$, $X\to c_{s\,i\,t\,e}$, $c_{s\,e\,x}\to y$, and $c_{s\,i\,t\,e}\to y$. (**a**) shows the results before any correction is performed, (**b**) shows the results of performing confound regression, and (**c**) and (**d**) show the results from counterbalancing by undersampling the majority class and oversampling the minority class, respectively. Statistical significance is obtained from 1,000 permutation tests and is shown with ** if p<0.01, * if p<0.05, and ‘n.s’ if p≥0.05.

Figure 2 shows the results of comparing different confound correction techniques for the ‘Binge’ phenotype. The following conclusions can be derived from this comparison:

1. Sex and site can confound the AAM analysis: As shown in subplot (a), all the input-output combinations involving the confounds ($X\to c_{s\,e\,x}$, $X\to c_{s\,i\,t\,e}$, $c_{s\,e\,x}\to y$ and $c_{s\,i\,t\,e}\to y$) produce significant prediction accuracies before any confound correction is performed. This further adds to the evidence that both the confounds $c_{s\,e\,x}$, $c_{s\,i\,t\,e}$ can strongly influence the accuracy of the main analysis X→y and confound the analysis. 2. Confound regression is not a good choice when followed by a non-linear ML method: Following confound regression, the results of $X\to c_{s\,e\,x}$ and $X\to c_{s\,i\,t\,e}$ should become non-significant as the signal *s*_*c*_ has been removed from X. However, it is seen that in some cases the non-linear models SVM-rbf and GB are capable of detecting the confounding signal *s*_*c*_ from the imaging data. The red arrow in the subplot (b) points out one such case in the example shown. This is not surprising as the standard confound regression removes linear components of the signal *s*_*c*_ but does not remove any non-linear components that might still be present in X (Görgen et al., 2018; Dinga et al., 2020). Furthermore, confound regression carries an additional risk of also regressing-out the useful signal in X that does not confound the analysis X→y but is a co-variate of both c and y (Dinga et al., 2020). 3. Counterbalancing with oversampling is the best choice for this study: As expected, counterbalancing forces the $c_{s\,e\,x}\to y$ and $c_{s\,i\,t\,e}\to y$ accuracies to chance-level by removing the correlation between c∼y (subplots c and d). It can be seen that after the undersampled counterbalancing the results of the main analysis X→y also become non-significant as indicated by the red arrow in (c). This drastic reduction in performance is likely due to the reduction in the sample size of the training data by n∼100-250 from undersampling. Therefore, counterbalancing with oversampling of the minority group is a better alternative compared to undersampling.

This comparison was also repeated for two other AAM phenotypes - ‘Combined-seo’ and ‘Binge-growth’ and the above findings were found to be consistent across all of them. Hence, counterbalancing with oversampling is used as the confound-control technique in the main analysis. When performing over-sampled counterbalancing, it is ensured that the oversampling is done only for the training data.

### ML exploration

**Figure 3.**
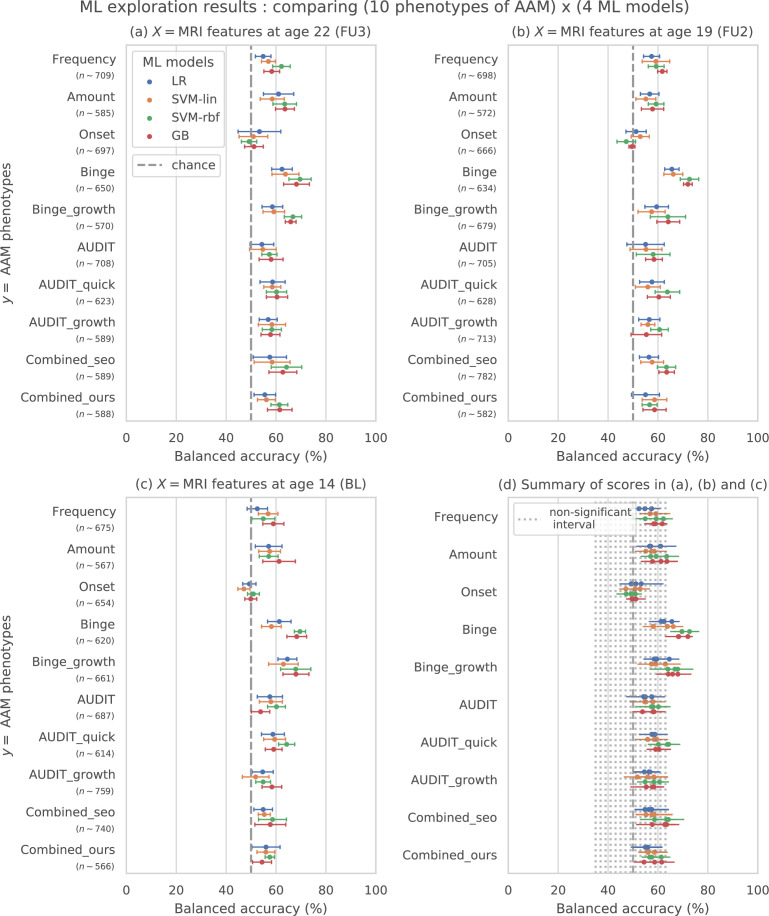
Results of the ML exploration experiments: The ten phenotypes of AAM tested are listed on the y-axis and the four ML models are represented with different color coding as shown in the legend of figure (**a**). For a given AAM label and ML model, the point represents the mean balanced accuracy across the 7-fold CV and the bars represent its standard deviation. Figure (**a**) shows the results when the imaging data from age 22 (FU3) is used, figure (**b**) shows results for age 19 (FU2) and figure (**c**) for age 14. Figure (**d**) shows the results from all three time point analyses in a single plot along with the interval of the balanced accuracy that were non-significant (p≤0.05) when tested with permutation tests.

The results from the ML exploration experiments are summarised in Figure 3. For the different AAM phenotypes, the balanced accuracies range between 45 and 73%. It must be noted that the results across different phenotypes are not directly comparable as each AAM phenotype classification task has a different sample size varying between ≈620-780 (refer to ‘Materials and methods’ Table 2 and Appendix 1—table 2 for the list of phenotypes and their respective sample size). These differences in the number of samples in the two classes AAM and controls could add additional variance in the accuracy. Nevertheless, some useful observations can be made from the consistenties found across the three time point analyses, depicted in subplots (a), (b), and (c) of Figure 3:

**Figure 3—figure supplement 1.**
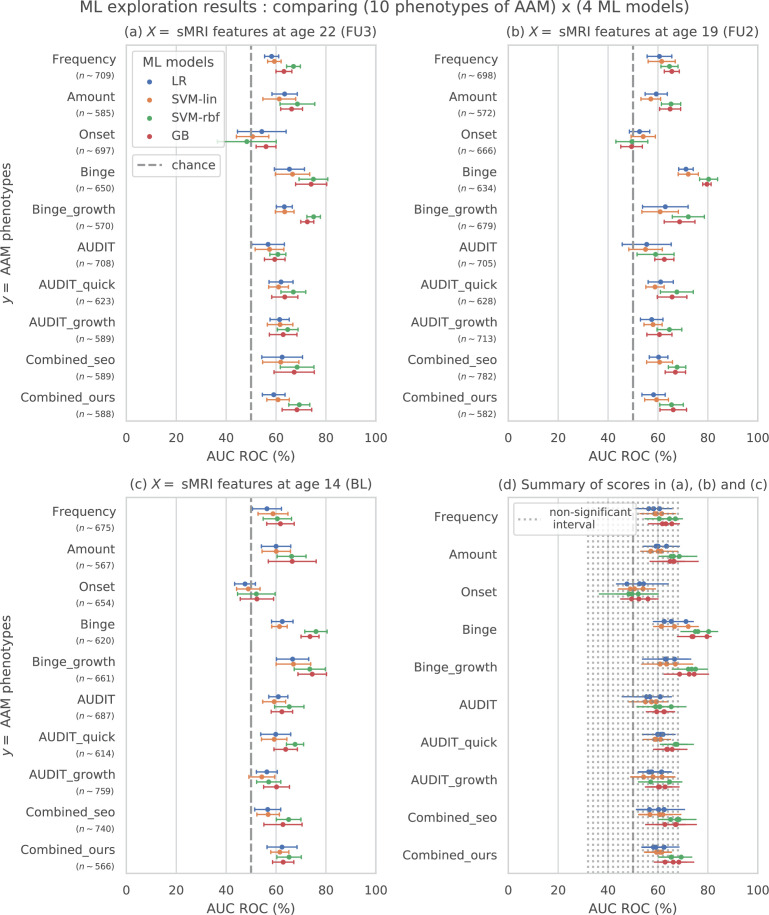
ML exploration results shown with AUC-ROC metric.

1. The most predictable phenotype from structural brain features for all three time point analyses is ‘Binge’ which measures the total lifetime experiences of being drunk from binge drinking.
2. Other individual phenotypes such as the amount of alcohol consumption (Amount), frequency of alcohol use (Frequency) and the age of AAM onset (Onset) are harder to predict from brain features compared to the binge drinking phenotype. The results on ‘Combined-seo’ and ‘Combined-ours’ shows that using phenotypes measuring amount and frequency of drinking in combination with binge drinking seems to also be detrimental to model performance.
3. All models perform poorly at predicting AAM phenotypes derived from AUDIT. This is surprising as AUDIT is considered a de facto screening test for measuring alcohol misuse (Kranzler and Soyka, 2018).
4. Among the four ML models, the SVM with non-linear kernel SVM-rbf, and the ensemble learning method GB perform better than the linear models LR and SVM-lin. This is further evident in the summary plot (d) in the figure.

**Table 2.** 10 phenotypes of Adolescent Alcohol Misuse (AAM) are derived and compared in this analysis. A description of each phenotype is provided here along with the link to the IMAGEN questionnaires ID used to generate the phenotype.

No.	Phenotype	Description	Questionnaire
1	Frequency	Number of occasions drinking alcohol in last 12 months	ESPAD 8b.
2	Amount	Number of alcohol drinks consumed on atypical drinking occasion	ESPAD prev31,AUDIT q2.
3	Onset	Had one or more binge-drinking experiences by the age of 14	ESPAD 29d
4	Binge	Total drunk episodes from binge-drinking in lifetime (by age 22)	ESPAD 19a,AUDIT q3.
5	Binge-growth	Longitudinal trajectory of binge-drinking experiences had per year	Growth curveof ESPAD 19b.
6	AUDIT	AUDIT screening test performed at the year of scan	AUDIT-total (q1-10).
7	AUDIT-quick	Only the first 3 questions of AUDIT screening test	AUDIT-freq (q1-3).
8	AUDIT-growth	Longitudinal changes in the AUDIT score measured over the years	Growth curve ofAUDIT-total.
9	Combined-seo	A combined risky-drinking phenotype from Seo et al., 2019 generated using amount, frequency, and binge-drinking data	ESPAD 8b, 17b, 19b,and TLFB alcohol2
10	Combined-ours	A combined risky-drinking phenotype developed by clusteringamount, frequency, and binge-drinking trajectory	AUDIT q1, q2,ESPAD 19a, growthcurve of ESPAD 19b.

In summary, the non-linear ML models SVM-rbf and GB coupled with the ‘Binge’ phenotype consistently perform the best in all three time point analyses. This is more clearly visible in the summary figure (d) where the results from all three analyses are combined in a single plot. Similar general observations can be made when the AUC-ROC metric is used to measure model performance (see Figure 3—figure supplement 1).

### Generalization

**Figure 4.**
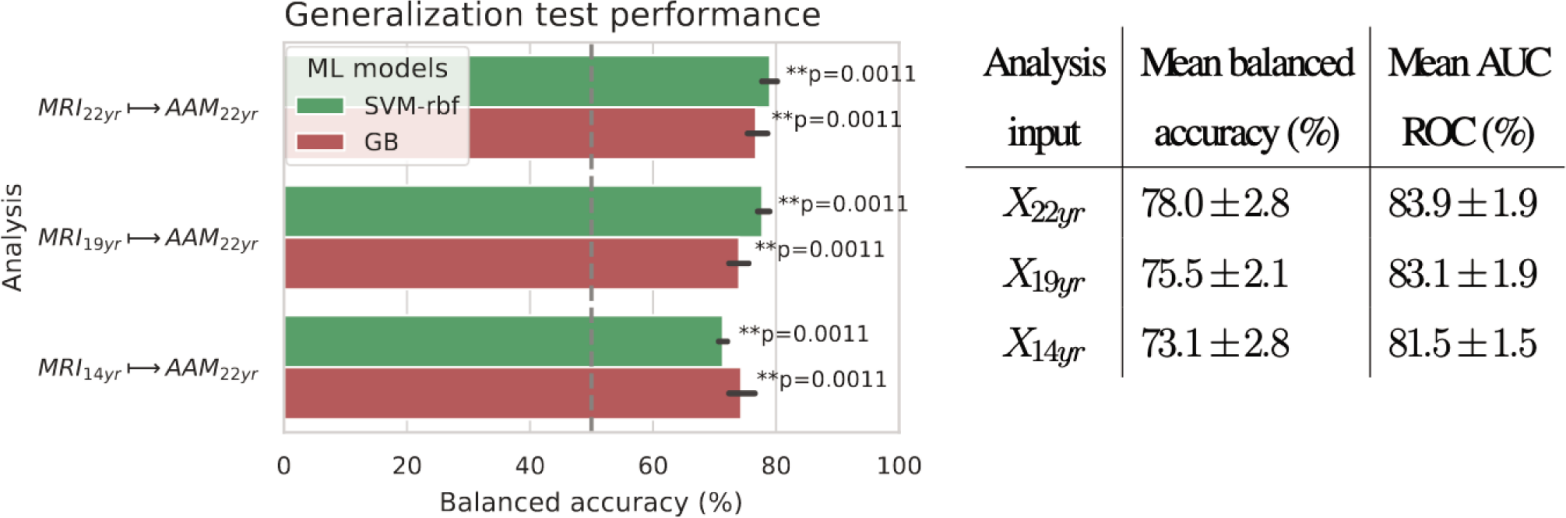
Final results for the three time point analyses on the ‘Binge’ drinking AAM phenotype obtained with the two non-linear ML models, kernel-based support vector machine (SVM-rbf) and gradient boosting (GB). The figure shows the mean balanced accuracy achieved by each ML model within each analysis while the table lists the combined average scores for each analysis. The ML models are retrained seven times on data ${}_{e\,x\,p\,l\,o\,r\,e}$ with different random seeds and evaluated on data ${}_{h\,o\,l\,d\,o\,u\,t}$ to obtain an estimate of the accuracy with a standard deviation. Statistical significance is obtained from 1000 permutation tests and is shown with ** if p<0.01, * if p<0.05, and ‘n.s’ if p≥0.05.

The generalization test is performed with ‘Binge’ phenotype as the label and the two non-linear ML models, SVM-rbf and GB. The final results are shown in Figure 4. For the three analyses using imaging data from age 22, age 19, and age 14, as input, an average balanced accuracy of 78%, 75.5%, and 73.5% are achieved, respectively. Their average ROC-AUC scores are 83.93%, 83.1%, and 81.5% for the respective analyses. The accuracies for all three time point analyses are significant with p<0.01. To get a better intuition, please refer to Figure 4—figure supplement 1 that shows the model accuracies against the accuracies obtained from permutation tests.

**Figure 4—figure supplement 1.**
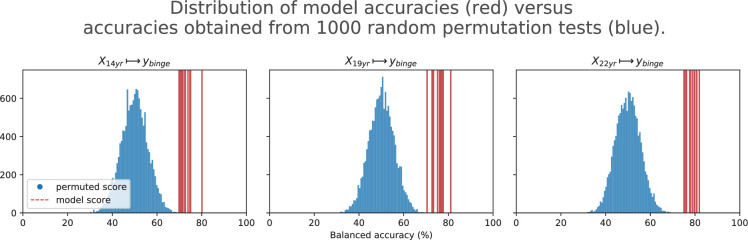
Visualization of the permutation test results.

To further assess the causality in the $M\,R\,I_{a\,g\,e\,14}\to A\,A\,M_{a\,g\,e\,22}$ analysis, we repeated it by using only subjects who had no binge drinking experiences by age 14 (n=477) and also with subjects who had a maximum of one binge drinking experience (n=565) by age 14. The balanced accuracy obtained on the holdout set was 72.9±2% and 71.1±2.3%, respectively.

### Important brain regions

**Figure 5.**
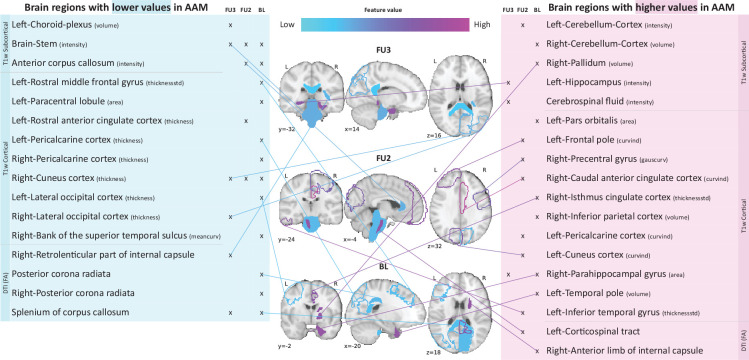
Most informative structural features for SVM-rbf model’s predictions on data ${}_{h\,o\,l\,d\,o\,u\,t}$. Most important features are listed and their locations are shown on a template brain for a better intuition for each of the three time point analyses. The features are color coded to also display whether these features have lower-than-average or higher-than-average values when the model predicts alcohol misusers. This figure is only illustrative and an exhaustive list of all informative features with their corresponding SHAP values are given in the Appendix 1—table 3. (Acronyms:: AAM: adolescence alcohol misuse, area: surface area, volume: gray matter volume, thickness: average thickness, thicknessstd: standard deviation of thickness, intensity: mean intensity, meancurv: integrated rectified mean curvature, gauscurv: integrated rectified gaussian curvature, curvind: intrinsic curvature index).

Following the generalization test, the most informative structural brain features are determined for the SVM-rbf model, as it performs relatively better among the two non-linear models tested on data ${}_{h\,o\,l\,d\,o\,u\,t}$ (see Figure 4). Figure 5 shows the list of the most important features for all three time point analyses and illustrates where they are located in the brain. It also shows whether these features have lower-than-average or higher-than-average values when the ML model predicts the subjects as AAMs.

Several clusters of regions and feature values can be identified. Most of the important subcortical features are located around the lateral ventricles and the third ventricle and include CSF-related features such as the CSF mean-intensity, volume of left choroid plexus, and left corticospinal tract in the brain stem. Several white matter tracts are found to be informative such as parts of the corpus callosum, internal capsule, and posterior corona radiata. Furthermore, all of these white matter tracts, along with the brain stem have lower-than-average intensities in AAM predictions. The prominent cortical features are spread across the occipital, temporal, and frontal lobes. In the $M\,R\,I_{a\,g\,e\,22}\to A\,A\,M_{a\,g\,e\,22}$ analysis important cortical features appear in the occipital lobe. In contrast, for the future prediction analyses $M\,R\,I_{a\,g\,e\,19}\to A\,A\,M_{a\,g\,e\,22}$ and $M\,R\,I_{a\,g\,e\,14}\to A\,A\,M_{a\,g\,e\,22}$, clusters appear in the limbic system (parts of the cingulate cortex and right parahippocampal gyrus), frontal lobe (left-pars orbitalis, left-frontal pole, right-precentral gyrus, and left-rostral middle frontal gyrus) as well as in the temporal lobe (left-inferior temporal gyrus, left-temporal pole, and right-bank of the superior temporal sulcus). In the occipital lobe, AAMs predictions have lower grey matter thickness in the right-cuneus, lateral occipital, and pericalcarine cortices, and higher curvature index in left-cuneus and left-pericalcarine cortex. The list of all the informative features are provided in Appendix 1—table 3 along with their feature type, modality, and respective SHAP values in each CV folds.

### Cross-site experiment

**Figure 6.**
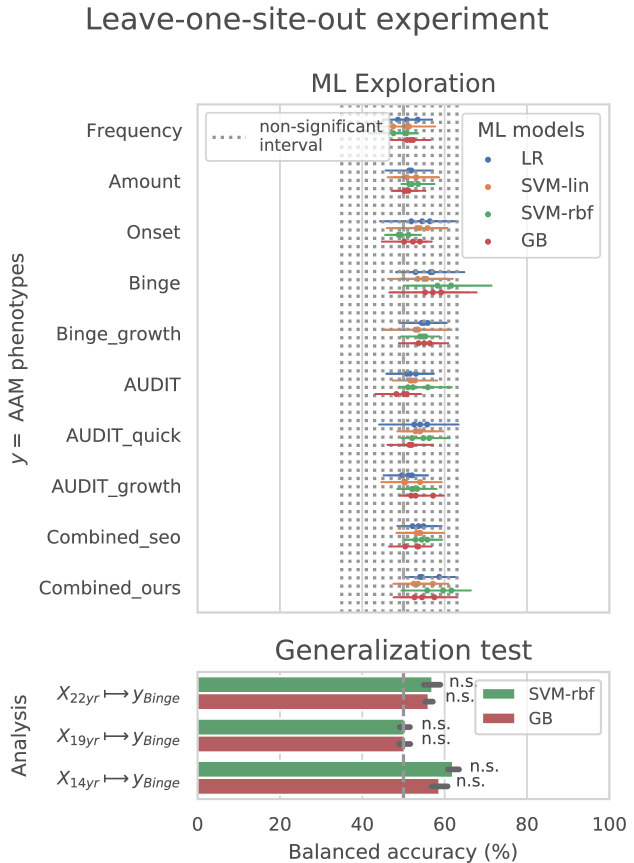
Analysis repeated with leave-one-site-out cross validations (CV).

The result from the *leave-one-site-out* CV experiment are shown in Figure 6. The ML models perform close-to-chance for all AAM labels in the ML exploration experiments and fail to produce a significant performance for any of the three time points in the generalization test. For the ‘Binge’ label in the ML exploration stage, the model accuracy displays very high variance, as compared to the main experiment (compare Figure 6 with Figure 3 (d)). This suggests that the performance of the ML models varies greatly across sites in this study.

## Discussion

For over two decades, researchers have tried to uncover the relationship that exist between adolescent alcohol misuse (AAM) and brain development. Many previous studies found that such a relationship exists (see Table 1) but with low-to-medium effect size (Nagel et al., 2005; Whelan et al., 2014; Squeglia et al., 2017; Seo et al., 2019; De Bellis et al., 2005; McQueeny et al., 2009; Luciana et al., 2013). The brain regions linked with AAM varied greatly across studies (see highlighted text in Table 1). This inconsistency in findings and effect sizes could be due to methodological limitations, small sample studies, unavailability of long-term longitudinal data like IMAGEN (Mascarell Maričić et al., 2020), or simply due to the heterogeneous expression of AAM in the brain. In our study, ML models predicted AAM with significantly above-chance accuracies in the range 73.1%-78% (ROC-AUC in 81.5%-83.9%) from adolescent brain structure captured at ages 14, 19, and 22. Thus, our results demonstrate that adolescent brain structure is indeed associated with alcohol misuse during this period.

The causality of the relationship between adolescent brain structure and AAM is not clear (Whelan et al., 2014; Robert et al., 2020). The relationship could arise from alcohol misuse inducing neurotoxicity (Zahr and Pfefferbaum, 2017) causing the observed changes in their brains. It could also be that these structural differences precede AAM and such adolescents are just more vulnerable towards alcohol misuse (Chambers et al., 2003; Sanchez-Roige et al., 2019). Such neuropsychological predisposition could stem from genetic predispositions or from influencing environmental factors such as early stress or childhood trauma (Baker et al., 2013; Ross et al., 2021), misuse of other drugs such as cannabis (French et al., 2015) and tobacco, and parental drug misuse (Jones and Nagel, 2019). There might also be an interaction effect between alcohol-induced neurotoxity and environmental and genetic predispositions (Robert et al., 2020). While the direction of causality is still under active investigation (Robert et al., 2020; Bourque et al., 2016), the significantly high accuracies obtained in our study for $M\,R\,I_{a\,g\,e\,19}\to A\,A\,M_{a\,g\,e\,22}$ and especially $M\,R\,I_{a\,g\,e\,14}\to A\,A\,M_{a\,g\,e\,22}$ suggest that these structural differences might be preceding alcohol misuse behavior. Out of the 265 subjects that took the ESPAD survey at age 14 and belonged to the AAM category in $M\,R\,I_{a\,g\,e\,14}\to A\,A\,M_{a\,g\,e\,22}$ analysis, 83.3% of subjects reported having no or just one binge drinking experience until age 14. When we repeated the $M\,R\,I_{a\,g\,e\,14}\to A\,A\,M_{a\,g\,e\,22}$ analysis with only the subjects who had no binge drinking experiences (n=477) or a maximum of one binge drinking experience (n=565) by age 14, we obtained a balanced accuracy of 72.9±2% and 71.1±2.3% respectively, on the holdout data. This is comparable to the main result of 73.1±2%. This result provides further evidence for the findings of Robert et al., 2020 that certain cerebral predispositions might precede alcohol abuse in adolescents. Thus, like (Robert et al., 2020) we also advocate caution when interpreting the results from previous cross-sectional studies suggesting alcohol-induced brain atrophy. We identified the most informative brain features for the ML predictions using SHAP that has been successfully applied to medical data (Lundberg and Lee, 2017; Lundberg et al., 2020; Molnar, 2022). The important features were found to be distributed across several subcortical and cortical regions of the brain, implying that the association between AAM and brain structure is widespread and heterogeneous. In accordance with previous studies, AAM was associated with lower DTI-FA intensities in several white matter tracts and the brain stem (McQueeny et al., 2009; Jacobus et al., 2013; Jones et al., 2018) and reduced GM thickness (Squeglia et al., 2017; Pfefferbaum et al., 2018), especially in the occipital lobe. Features of anterior cingulate cortex (Squeglia et al., 2017; Seo et al., 2019; Jones et al., 2018), middle frontal and precentral gyrus (Luciana et al., 2013), hippocampus (De Bellis et al., 2000; Nagel et al., 2005), and right parahippocampal gyrus (Whelan et al., 2014) were also found to be informative, although the type of feature and the average feature value in AAMs differed from previous studies. Features from the frontal lobe and cerebellum were informative only for future AAM (Jones and Nagel, 2019) but not for current AAM prediction, in contrast to findings of De Bellis et al., 2005; Whelan et al., 2014; Seo et al., 2019. This difference could be due to the meticulous confound control performed in this study for sex and site of the subjects. Additionally, our ML models also found CSF-related features in the third and lateral ventricles, and some regions of the temporal cortex as informative features for AAM prediction.

In the ML exploration stage, we found that the binge drinking phenotype, which is commonly used in previous studies (Nagel et al., 2005; Whelan et al., 2014; Robert et al., 2020), was the most predictable phenotype of AAM as compared to frequency, amount, or onset of alcohol misuse. Curiously, phenotypes derived from AUDIT, which is a gold standard of screening for alcohol misuse (Kranzler and Soyka, 2018), did not score significantly above-chance in any of the three time point analyses. Other similar compound metrics that use measures of alcohol use frequency and amount along with binge drinking, such as ‘Combined-seo’ and ‘Combined-ours’, also perform worse than using just the binge drinking information. This suggests that using other phenotypes of alcohol misuse in combination with binge drinking was detrimental to the prediction task, as compared to using only binge drinking. Different phenotypes of AAM capture slightly different psychosocial characteristics of adolescents (Castellanos-Ryan et al., 2013). For instance, ‘Amount’ correlates significantly with agreeableness and a life history of relocation valence (r=-0.14, p<0.001), accident valence (r=-0.16, p<0.001) and sexuality frequency (r=-0.17, p<0.001), whereas the other phenotypes do not (p>0.01). ‘AUDIT’ and it’s derivatives significantly correlate with impulsivity trait (r=0.23, p<0.001) on SURPS, where as ‘Binge’ does not (r=0.09, p>0.01) but they both correlate with sensation seeking trait (r>0.29, p<0.001) as also found in previous studies (Castellanos-Ryan et al., 2011). Castellanos-Ryan et al., 2013 have found that these two traits manifest differently in the brain. Therefore, one can hypothesize that the psychosocial differences and their associated neural correlates (Castellanos-Ryan et al., 2011) between ‘Binge’ and the other AAM phenotypes might explain the 2-10% higher accuracy obtained with ‘Binge’.

In contrast to the main results, the ML models failed to attain significantly high prediction accuracy in the *leave-one-site-out* experiment as the scores displayed high variance across the CV folds (refer to Figure 6). On further investigation, we found that the ML models performed especially poorly on test data from Dublin and Nottingham (≤60% balanced accuracy) across all time points and metrics. On the contrary, models always performed better-than-chance on subjects from Dresden, Mannheim, and Hamburg. When we compared this with the main experiment, a similar pattern was found. The models least generalized to test subjects from the sites Dublin and Nottingham, across all 7 CV folds. Notably, the accuracy across sites did not correlate with the sample size of the sites, the ratio of AAMs to controls in the site, or their sex distribution. The results are shown in Appendix 1—figure 2 and Appendix 1—figure 3. Altogether, these results suggest that the relationship discovered in this study performs diversely on subjects from different sites and does not generalize equally across all sites of the IMAGEN dataset.

Methodological insights: To the best of our knowledge, this is the first study to analyze and reports results on the complete longitudinal data from IMAGEN, including the follow-up 3 data. Two previous studies, (Whelan et al., 2014; Seo et al., 2019) performed similar ML analysis on the IMAGEN data and unlike us, found only a weak association between structural imaging and AAM. The logistic regression model in Whelan et al., 2014 scored 58±8% ROC-AUC when predicting AAM at age 14 from structural imaging features collected at age 14 (BL) and 63±7% ROC-AUC at predicting AAM at age 16 (FU1). This lower accuracy with high variance obtained in their experiments can be attributed to - (a) the relatively smaller sample size used in their study (n∼265-271), (b) unavailability of long-term AAM information from IMAGEN’s FU2 and FU3 data, (c) using only a linear ML model, and (d) only using GM volume and thickness as structural features. On the other hand, Seo et al., 2019’s models achieved accuracies in the range 56-58% when predicting AAM at age 19 (FU2) using imaging features from age 19, and did not get a significant accuracy when they used imaging features from age 14. This lower performance can be attributed to the following experimental design decisions - (a) Seo et al., 2019 used GM volume and thickness features from just 24 regions of the brain associated to cue-reactivity, (b) their AAM phenotype is not the best phenotype of AAM as evident from the results of our ML exploration (see results for ‘Combined-seo’ in Figure 3), and (c) the confound-control technique used in their study, confound regression, can result in under-performance as demonstrated in Figure 2.

In contrast to these previous works, our study has the following advantages: First, we use 719 structural features extracted from 2 MRI modalities, T1w and DTI, that include not only GM volume and thickness but also surface area, curvature, and WM and GM intensities from all cortical and sub-cortical regions in the brains. Second, we empirically derive the best AAM label for the task by comparing different phenotypes previously used in the literature. For the different AAM phenotypes, the balanced accuracies range between chance to significant performance (45%-73%), emphasizing the importance of the choice of the label in such ML studies with low effect sizes. And finally, we test different confound correction techniques and use the one that effectively controls for the influence of confounds without also destroying the signal of interest. In summary, the higher accuracy in the current study can be attributed to not just the availability of long-term data on AAM but also to the rigorous comparison of different labels of AAM, different ML models and confound control techniques.

Among the four different ML models tested, the two non-linear models, SVM-rbf and GB, consistently performed better than the two linear models. We also explicitly ensured that the confounding influence of sex and site were eliminated by combining suggestions from Görgen et al., 2018 and Snoek et al., 2019. We found evidence that the linear confound regression technique used often in previous ML-based neuroimaging studies (Seo et al., 2019; Robert et al., 2020; Snoek et al., 2019), might not be the best choice as it cannot be used with non-linear models such as SVM-rbf or Naive Bayes used in Seo et al., 2019 and distorts the signal of interest from the neuroimaging data (Dinga et al., 2020) as seen in Figure 2. In contrast, counterbalancing using oversampling is recommended as it successfully removed the influence of the confounds without reducing the sample size in the study.

Future work: An important follow-up work would involve further investigating the association we found between AAM and adolescent brain structure and its clinical implications. For instance, one can analyze if certain environmental risk factors such as childhood abuse, parental drug use, or life event stressors mediate the relationship we found between brain structure and AAM behavior in the IMAGEN cohort. Another direction would be to further investigate the brain features associated with AAM and understand the relative contributions of specific brain networks (for example, similar to Seo et al., 2019) and certain specific feature types such as thickness, or volumes. Specifically, since ML feature attribution methods such as SHAP can be misled by the presence of correlated features (Molnar, 2022; Lundberg and Lee, 2017), it would be necessary to before-hand determine which features might be correlated and either exclude them, or permute correlated features together in groups when computing SHAP values (Molnar, 2022). Another important future work would be to reproduce our findings on another data set such as NCANDA (Brown et al., 2015) comprising adolescent subjects from a different geographic region. It would also be interesting to explore other modalities such as functional connectivity (fMRI) to predict AAM (Ruan et al., 2019).

### Conclusion

This study analyzed alcohol misuse in adolescents and their brain structure in the large, longitudinal IMAGEN dataset consisting of n∼1182 healthy adolescents (Schumann et al., 2010; Mascarell Maričić et al., 2020). We found that alcohol misuse in adolescents can be predicted from their brain structure with a significant and high accuracy of 73%-78%. More importantly, alcohol misuse at age 22 could be predicted from the brains at age 14 and age 19 with significant accuracies of 73.1% and 75.55%, respectively. This suggests that the structural differences in the brain might at least partly be preceding alcohol misuse behavior (Robert et al., 2020). Results of a *leave-one-site-out* experiment also revealed that the relationship discovered by the ML models may not generalize to all the sites in the IMAGEN dataset equally, particularly, to subjects from the sites Nottingham and Dublin. We extensively compared different phenotypes of alcohol misuse such as frequency of alcohol use, amount of use, the onset of alcohol misuse, and binge drinking occasions and found that binge drinking is the most predictable phenotype of alcohol misuse. We also compared four different ML models and found that the two non-linear models - SVM-rbf and GB - perform better than the two linear models, SVM-lin and LR. We also evaluated different confound-control techniques and found that counter-balancing with oversampling is most beneficial for the current task. To the best of our knowledge, this was the first study to analyze and report results on the follow-up 3 data from IMAGEN. The results of our exploratory study advocate that collecting long-term, large cohorts of data, representative of the population, followed by a systematic ML analysis can greatly benefit research on complex psychiatric disorders such as AUD.

## Materials and methods

### Data

**Figure 7.**
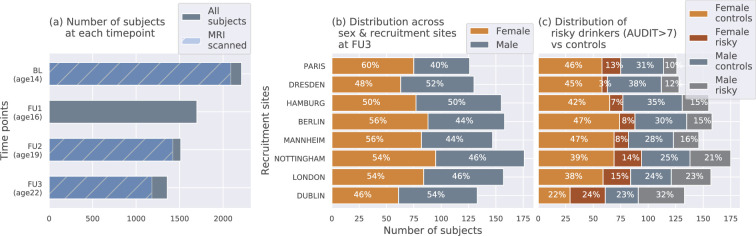
The IMAGEN dataset: (**a**) Data is collected longitudinally at 4 stages of adolescence - age 14 or baseline (BL), age 16 or follow-up 1 (FU1), age 19 or follow-up 2 (FU2) and, finally age 22 or follow-up 3 (FU3). The blue bar shows the number of subjects with brain imaging data. (**b**) The distribution of subjects across sex and the site of recruitment, for the 1182 subjects that were scanned at FU3 (**c**) The same distribution across sex and site also showing the proportion of subjects that meet the AUDIT ’risky drinkers’ category at FU3.

The IMAGEN dataset (Mascarell Maričić et al., 2020; Schumann et al., 2010) is currently one of the best candidates for studying the effects of alcohol misuse on the adolescent brain. Most large-sample studies listed in Table 1 [27, 29 and 30] used the IMAGEN dataset for their analysis. It consists of data collected from over 2000 young people and includes information such as brain neuroimaging, genomics, cognitive and behavioral assessments, and self-report questionnaires related to alcohol use and other drug use. The data was collected from 8 recruitment centers across Europe, at 4 successive time points of adolescence and youth. Figure 7 (a) shows the number of subjects at each time point and the number of participants that were scanned. Subjects were not scanned in FU1. More details regarding recruitment of subjects, acquisition of psychosocial measures, and ethics can be found on the IMAGEN project website (https://imagen-europe.com/standard-operating-procedures). Written and informed consent was obtained from all participants by the IMAGEN group and the study was approved by the institutional ethics committee of King’s College London,University of Nottingham, Trinity College Dublin, University of Heidelberg, Technische Universität Dresden, Commissariat à l’Energie Atomique et aux Energies Alternatives, and University Medical Center at the University of Hamburg in accordance with the Declaration of Helsinki (Association, 2013).

#### Structural neuroimaging data

**Figure 8.**
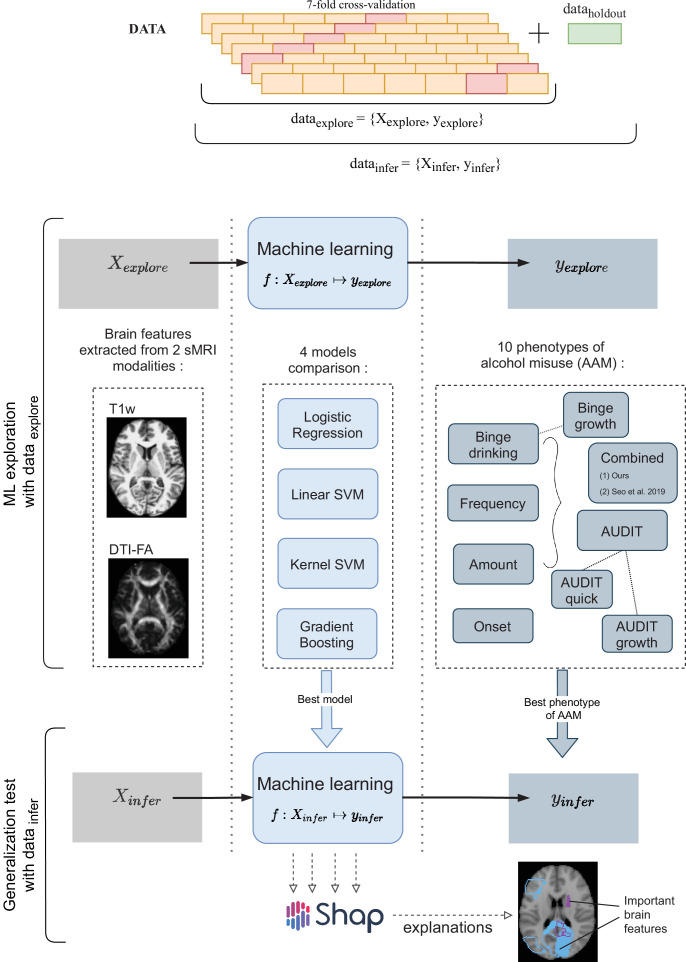
A schematic representation of the experimental procedure followed for all 3 time point analyses. In the ML exploration stage, we experiment with four ML models and 10 phenotypes of AAM on 80% of the data (data ${}_{e\,x\,p\,l\,o\,r\,e}$) using a sevenfold cross-validation scheme. Once the best ML model, the best phenotype of AAM, and the most appropriate confound-control technique are determined, the *generalization test* is performed on data ${}_{i\,n\,f\,e\,r}$ by using the data ${}_{h\,o\,l\,d\,o\,u\,t}$ subset as the test data. The result from the generalization test are reported as the final results and the informative brain features are determined at this stage using SHAP (Lundberg and Lee, 2017).

To investigate the effects of alcohol on brain structure, two MRI modalities have been used predominantly in the literature - (a) T1-weighted imaging (T1w), and (b) Diffusion Tensor Imaging (DTI) (see Table 1). While T1w MRI can be used to derive general features of the brain structure such as cortical and sub-cortical volumes, areas, and gray-matter thicknesses, DTI captures white matter microstructures by probing water molecule motion. An axial slice (z=80) of both of these MRI modalities of a control subject from the IMAGEN data are shown in Figure 8. Both modalities were recorded using 3-Tesla scanners. The T1w images were collected using sequences based on the ADNI protocol (Wyman et al., 2013). The IMAGEN consortium used Freesurfer’s recon-all pipeline to process these images and extract structural features. This involves registering the T1w-images to the Talairach template brain, automatic extraction of gray matter, white matter and cerebrospinal fluid (CSF) sections, and then segmenting them into 34 cortical regions per hemisphere and 45 sub-cortical regions.The grey matter volume (in mm^3^), surface area (in mm^2^), thickness (in mm), and surface curvature, are extracted for each of the cortical regions using the Desikan-Killiany atlas, along with global features such as total intracranial, total grey matter, white matter and CSF volumes. For the subcortical regions, the mean intensity and volume are determined. This results in a total of 656 structural features per subject. DTI scans were acquired using the protocol described in Jones et al., 2002 and Fractional Anisotropy (FA) is derived from the DTI using FMRIB’s Diffusion Toolbox FDT. The DTI-FA images are then non-linearly registered to the MNI152 space (1 mm^3^) and the average FA intensity at 63 regions with white matter tracts are calculated using the TBSS toolbox (Smith et al., 2006) by the IMAGEN consortium (https://github.com/imagen2/imagen_processing/tree/master/fsl_dti). Subjects with FA intensity greater than 3 standard deviations from the mean are excluded as outliers.

#### Alcohol misuse phenotypes

Information related to alcohol use and misuse can be found in the AUDIT screening test (*AUDIT questionnaire (link*)) (Alcohol Use Disorder Identification Test), ESPAD questionnaire (European School Survey Project on Alcohol and other Drug), and the TLFB logs (Timeline-Followback Interview). Previous studies used different metrics of alcohol misuse such as the number of binge drinking episodes (Squeglia et al., 2012; Whelan et al., 2014; Jones and Nagel, 2019; Robert et al., 2020), the frequency and amount of alcohol consumption (Squeglia et al., 2015; Pfefferbaum et al., 2018; Kühn et al., 2019; Seo et al., 2019; Sullivan et al., 2020), and even the age of onset of alcohol misuse (Ruan et al., 2019) to characterize AAM. There has not yet been a systematic comparison of these different phenotypes.

In this paper, we use four alcohol misuse metrics to derive ten phenotypes of AAM, (a) frequency of alcohol use, (b) amount of alcohol consumed per drinking occasion, (c) year of onset of alcohol misuse, and (d) the number of binge drinking episodes. These phenotypes are listed in Table 2 and include each of the individual metrics, their combinations, and their longitudinal trajectories from age 14–22. The longitudinal phenotypes, ‘Binge-growth’ and ‘AUDIT-growth’, are generated using latent growth curve models (Deeken et al., 2020) to capture the alcohol misuse trajectory over the four time points - BL, FU1, FU2, and FU3. To derive the AAMs group and the controls from each alcohol misuse metric, a standard procedure is followed that is similar to Seo et al., 2019; Ruan et al., 2019. First, the phenotype is used to categorize the subjects into three stages of alcohol misuse severity - heavy AAMs, moderate misusers, and safe users. Moderate misusers are then excluded from the analysis (≈250-400 subjects) and ML classification is performed with heavy misusers as AAMs and safe users as controls. Appendix 1—figure 1 and Appendix 1—table 2 shows how the subjects are divided into these three sub-groups for each of the 10 phenotype. Appendix 1—table 2 also lists the final number of subjects in each sub-group in the FU3 analysis, as an example. The data analysis procedure can be found in the project code repository (https://github.com/RoshanRane/ML_for_IMAGEN; Rane and Kim, 2022) within the *dataset-statistics* notebook.

#### Confounds in the dataset

Diagram (c) in Figure 7 shows how the proportion of risky alcohol users varies across the 8 recruitment sites and among the male and female subsets at each site within the dataset. For example, a greater portion of subjects from sites like Dublin, London, and Nottingham indulge in risky alcohol use compared to the sites from mainland Europe. Similarly, at most sites, a greater portion of males are risky alcohol users compared to females. These systematic differences can confound ML analyses since ML models can use the sex and site information present in the neuroimaging data to indirectly predict AAM, instead of identifying alcohol-related effects in the brain structure. This problem of confounds in multivariate analysis (Rao et al., 2017; Görgen et al., 2018; Snoek et al., 2019) and the methods used to control for its effects are explained in further detail in the next section.

### Methods

Three time point analyses are performed in this study. Each time point analysis is divided into two stages called the *ML exploration* stage and the *generalization test* stage. The ML exploration is performed with 80% of data (randomly sampled). The remaining 20% (n=147) serve as an independent test data, called the data ${}_{h\,o\,l\,d\,o\,u\,t}$, which is only used once, in the end, to perform the final inference and report the results. This design allows us to first determine the best ML algorithm for the task and the best phenotype of AAM, and then test the results on an independent subset of the data. Pseudocode of this pipeline is provided at the end of Appendix (Algorithm 1) and was implemented using python’s *scikit-learn* software package (https://scikit-learn.org/stable/about.html). The two-stage cross-validation (CV) with a inner n-fold cross-validation (CV) procedure is designed to prevent ‘double dipping’ (Vul et al., 2009; Kriegeskorte et al., 2009). All data preprocessing and analysis is executed only on the training data in data ${}_{e\,x\,p\,l\,o\,r\,e}$, and only applied on the test data during validation. This ensures that there are no data leakage issues that were found in several previous ML neuroimaging studies (Wen et al., 2020). Since multi-site data is used, another additional experiment is performed to test the ability of the ML models to generalize across recruitment sites. In this experiment, instead of randomly sampling 20% of the subjects for the data ${}_{h\,o\,l\,d\,o\,u\,t}$, all subjects from the Nottingham site (n=176) are set aside as data ${}_{h\,o\,l\,d\,o\,u\,t}$. Then, subjects from each of the remaining 7 recruitment sites are used as onefold in the sevenfold CV performed during the ML exploration phase. This method of CV is termed *leave-one-site-out* CV (Rozycki et al., 2018).

#### MRI features

The 656 morphometric features extracted from T1w sMRI modality and the 63 features extracted from the DTI-FA modality are used together as the input for the ML models at both stages. Each feature is standardized to have zero mean and unit variance across all subjects (mean and variance are estimated only on the training data, and then applied to the test data). Features with zero variance are dropped.

#### ML models

Four ML models are tested in this study. These include logistic regression (LR), linear SVM (SVM-lin) (Boser et al., 1992), kernel SVM with a radial basis function (KSVM-rbf) (Chapelle et al., 2002), and a gradient boosting (GB) classifier (Friedman, 2001). LR and SVM-lin are linear ML methods, whereas SVM-rbf and GB are capable of learning non-linear mappings. We use the liblinear (Fan et al., 2008) implementation of SVM-lin and XGBoost (Chen and Guestrin, 2016) implementation of GB. GB is an ensemble learning method. The hyperparameters of the models are tuned using an inner-CV and are listed in Appendix 1—table 1. Testing 4 different ML models helps to account for any modeling-related bias (Wolpert and Macready, 1997) in the final results. Combining the 4 ML models and the ten different phenotypes of AAM, we end up with a total of 40 ML classification runs in the ML exploration stage.

#### Evaluation metrics

The model performance is evaluated using the *balanced accuracy* metric (Urbanowicz and Moore, 2015). It is formulated as the mean of the model’s accuracies for each class (AAM and controls) in the classification. Therefore, it is insensitive to class imbalances in the data. Along with this, the area under the curve of the receiver-operator characteristic (AUC-ROC) is also calculated. In ML exploratory stage, seven measures are obtained for each metric from the outer sevenfold CV which helps to estimate mean of the model performance and get a sense of the variance (Bengio and Grandvalet, 2004). During generalization test, the ML models are retrained seven times on data ${}_{e\,x\,p\,l\,o\,r\,e}$ with different random seeds and reevaluated on data ${}_{h\,o\,l\,d\,o\,u\,t}$ to gain an estimate of the model performance on data ${}_{h\,o\,l\,d\,o\,u\,t}$. The statistical significance of the final generalization test accuracies is calculated using permutation testing (Ojala and Garriga, 2010). The permutation test is performed by running the entire ML pipeline with randomly shuffled labels in the training data, while keeping the labels in the test data fixed. This is repeated 1000 times to generate the null-hypothesis (*H*_0_) distribution and derive the p-value. Since three time point analyses are performed on the same subjects, Bonferroni correction is applied on the p-values to control for the false-positive rate from this multiple comparison.

#### Model interpretation

The associations learned by the ML models between structural brain features and AAM is extracted using a post-hoc feature importance attribution technique called SHAP (Lundberg and Lee, 2017). SHAP (SHapley Additive exPlanations) uses the concept of *Shapley Values* from cooperative game theory to fairly determine the marginal contribution of each input feature to model prediction (Lundberg and Lee, 2017). Among the several SHAP estimator types (Molnar, 2022), we use the permutation-based estimator as it is compatible with all 4 ML models used in this analysis.

Following the generalization test, a SHAP value ($S_{s,f}$) is generated for each input feature f of each subject s in data ${}_{h\,o\,l\,d\,o\,u\,t}$. The goal is to determine which of the 719 features were most informative for the model when classifying AAMs from controls. Feature importance can be determined by looking at the average absolute SHAP value of each feature across all subjects $\bar{S_{f}}=\frac{1}{N}\,\sum_{s=1}^{N}\left|S_{s,f}\right|$, where N denotes the total subjects in data ${}_{h\,o\,l\,d\,o\,u\,t}$. The most significant features are chosen as those features that have $\bar{S_{f}}$ value at least two times higher than the average SHAP value across all the features $\bar{S}=\frac{1}{719}\,\sum_{f=1}^{719}\left|\bar{S_{f}}\right|$. Our feature importance estimation can be confounded by the presence of correlated features (Molnar, 2022). When several features are correlated, the ML models might use only some features for its prediction and ignore the rest and this preferential bias can be reflected in the SHAP values. Since the generalization test is repeated seven times with different random seeds, we have seven instances of $\bar{S_{f}}$ available. Therefore, we repeat the SHAP estimation on each $\bar{S_{f}}$ with different random permutations and check for consistency of feature importance scores across these seven trials. Only the features that consistently have $\bar{S_{f}}\geq 2*\bar{S}$ across at least six of the seven runs are listed as the most informative features. Following this, it is determined if these informative features have higher-than-average or lower-than-average values when predicted as AAM. This information is further relevant for deriving clinical insights about how AAM brain structure differs from controls.

#### Correcting for confounds

In ML, a confounding variable c is defined as a variable that correlate with the target y and is deducible from the input X, and this relationship X→c→y is not of primary interest to the research question and hinders the analysis (Snoek et al., 2019). As demonstrated by the diagram on the right, a confounding variable c can form an alternative explanation for the relationship between X and y and distract the ML models from detecting the signal of interest *s*_*y*_ between X→y. In this study, the sex of the subjects and their site of recruitment can confound the AAM analysis (Seo et al., 2019) since they correlate with the output AAM labels and are predictable from the input structural brain features. Instead of detecting the effects of alcohol misuse in the brain *s*_*y*_, the ML models could potentially use the information about the confounds *s*_*c*_ to predict AAM along the alternative pathway (shown with the red dotted lines) and produce significant but confounding results (Seo et al., 2019; Snoek et al., 2019; Dinga et al., 2020). In neuroimaging studies, two methods have been extensively employed for correcting the influence of confounds:

1. *Confound regression*: In this method, the influence of the confounding signal *s*_*c*_ on X is controlled by regressing out the signal from features in X(Rao et al., 2017). This can remove the alternative confounding explanation pathway by eliminating the link *s*_*c*_ between X→c.
2. *Post hoc counterbalancing*: The correlation between the confound and the output c∼y can be removed by resampling the data after the data collection. This method potentially removes the alternative confounding pathway by abolishing the relationship c→y(Rao et al., 2017). The resampling is performed such that the distribution of the values of the confounding variable c is similar across all classes of y (AAM and controls). So for example, after counterbalancing for sex in this study, the ratio of male-to-female subjects should be the same in AAMs and controls. One common technique of counterbalancing for categorical confounds (e.g. sex, site) involves randomly dropping some samples from the larger classes in y until they are equal. This is called counterbalancing *with undersampling*. However, this will result in a reduction in the sample size and hence the statistical power of the study. Another way to counterbalance without losing samples involves performing *sampling-with-replacement* on the smaller classes in y. This is called counterbalancing *with oversampling*. One should take care that the sampling-with-replacement is done only on the training data, after the train-test split is performed.

To assess whether confound regression worked and the confounding signal *s*_*c*_ is removed successfully, a confound correction method recently proposed by Snoek et al., 2019 can be used. In this method, the ML algorithm used in the original analysis is reused to predict the confound c from the neuroimaging data X. Following a successful confound regression, the confound should not be predictable anymore from X and X→c should produce insignificant or chance accuracy. Similarly, to determine if counterbalancing was successful and the correlation c∼y was removed, we used the *Same Analysis Approach* by Görgen et al., 2018. Here, the same ML algorithm is used to predict the confound c from the labels y(Görgen et al., 2018). An above-chance significant prediction accuracy between c→y would indicate that the correlation c∼y still exists and the counterbalancing was not successful. Since the confounds $c_{s\,e\,x}$ and $c_{s\,i\,t\,e}$ are categorical, they are first one-hot encoded to ensure no false ordinal relationship is implied. The confound correction methods are only performed on the training data as recommended by Snoek et al., 2019. The balanced accuracy metric used ensures that we account for any class imbalances in the test data. Before starting the ML exploration, we first compare these different confound correction methods and choose the most suitable method among them.

**Table:** 

**Algorithm 1. Pipeline pseudocode:** Procedure followed for each of the 3 analyses. The ‘⊢’ operation represents fitting or training the ML model given on the left side of the operation on the data given on the right side:
$\left\{d\,a\,t\,a_{e\,x\,p\,l\,o\,r\,e},d\,a\,t\,a_{h\,o\,l\,d\,o\,u\,t}\right\}\subset d\,a\,t\,a_{i\,n\,f\,e\,r}$ ⊳ Keep aside 20% as *data_holdout_* Start exploratory analysis M∈ {LR, SVM-lin, SVM-rbf, GB} $y\in \left\{y_{f\,r\,e\,q},y_{a\,m\,o\,u\,n\,t},\ldots \,y_{b\,i\,n\,g\,e}\right\}$ ⊳ select one of 10 AAM phenotypes **for** $i_{o\,u\,t\,e\,r}\in \left\{1,2,\ldots ,7\right\}$ **do** ⊳ Split *data_explore_* into 7 equal outer folds $t\,r\,a\,i\,n_{o\,u\,t\,e\,r}\leftarrow \left\{d\,a\,t\,a_{e\,x\,p\,l\,o\,r\,e}\,\left[i\right]|i\neq i_{o\,u\,t\,e\,r}\right\}$ $t\,e\,s\,t_{o\,u\,t\,e\,r}\leftarrow \left\{d\,a\,t\,a_{e\,x\,p\,l\,o\,r\,e}\,\left[i\right]|i=i_{o\,u\,t\,e\,r}\right\}$ **for** P∈ℙ **do ** ⊳P is set of all hyperparameter combinations **for** $i_{i\,n\,n\,e\,r}\in \left\{1,2,\ldots ,5\right\}$ **do ** ⊳ Split train_outer_ into 5 equal inner folds $t\,r\,a\,i\,n_{i\,n\,n\,e\,r}\leftarrow \left\{t\,r\,a\,i\,n_{o\,u\,t\,e\,r}\,\left[i\right]|i\neq i_{i\,n\,n\,e\,r}\right\}$ $t\,e\,s\,t_{i\,n\,n\,e\,r}\leftarrow \left\{t\,r\,a\,i\,n_{o\,u\,t\,e\,r}\,\left[i\right]|i=i_{i\,n\,n\,e\,r}\right\}$ $M\,\left(P\right)⊧t\,r\,a\,i\,n_{i\,n\,n\,e\,r}$ $a\,c\,c_{i}=$ evaluate $\left(M\,\left(P\right),t\,e\,s\,t_{i\,n\,n\,e\,r}\right)$ **end for** $a\,c\,c_{P}=$ mean $\left(a\,c\,c_{i}|\forall i_{i\,n\,n\,e\,r}\right)$ ⊳ average accuracy for hyperparameter combination *P* **end for** $\hat{P}\leftarrow \{P|$ highest $\left(acc_{P}|P\in P)\right\}$ $M\,\left(\hat{P}\right)⊧t\,r\,a\,i\,n_{o\,u\,t\,e\,r}$ $a\,c\,c_{j}=$ evaluate $\left(M\,\left(\hat{P}\right),t\,e\,s\,t_{o\,u\,t\,e\,r}\right)$ **end for** $a\,c\,c_{\left(M,y\right)}=$ mean $\left(a\,c\,c_{j}|\forall i_{o\,u\,t\,e\,r}\right)$ ⊳ average accuracy for model M and label y $\hat{M},\hat{y}\leftarrow \{M|$ highest $\left(a\,c\,c_{\left(M,y\right)}|\forall \left(M,y\right)\right)$ ⊳ select the best model $\hat{M}$ and AAM phenotype $\hat{y}$ **Start** generalization test $\hat{M}\,\left(\hat{P}\right)⊧d\,a\,t\,a_{e\,x\,p\,l\,o\,r\,e}$ a⁢c⁢c= evaluate $\left(\hat{M}\,\left(\hat{P}\right),d\,a\,t\,a_{h\,o\,l\,d\,o\,u\,t}\right)$

## Figures and Tables

**Appendix 1—figure 1.**
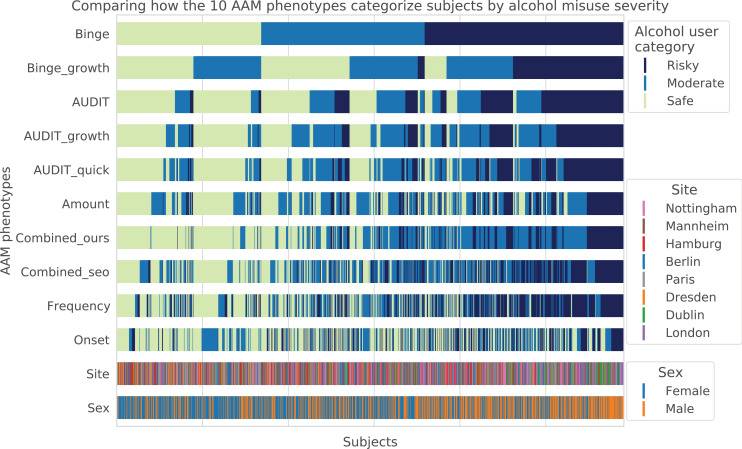
Visualizing AAM phenotype categorization: A qualitative comparison showing how the ten AAM phenotypes categorize the same subjects into the three alcohol user classes – risky alcohol users, moderate users, and safe or non-users. Each color-coded vertical line in the diagram represents one subject, out of the total 1182 subjects. It can be observed that the Frequency, Onset, and Amount phenotypes categorize very differently from Binge, showing that they capture different factors of alcohol misuse. All AUDIT-derived phenotypes are similar to each other but are different from the Binge phenotype. Furthermore, sex and site-specific variations can be detected. For instance, more males appear on the ‘risky’ groups compared to females. Similarly, most subjects from Dublin are clustered on the risky side.

**Appendix 1—figure 2.**
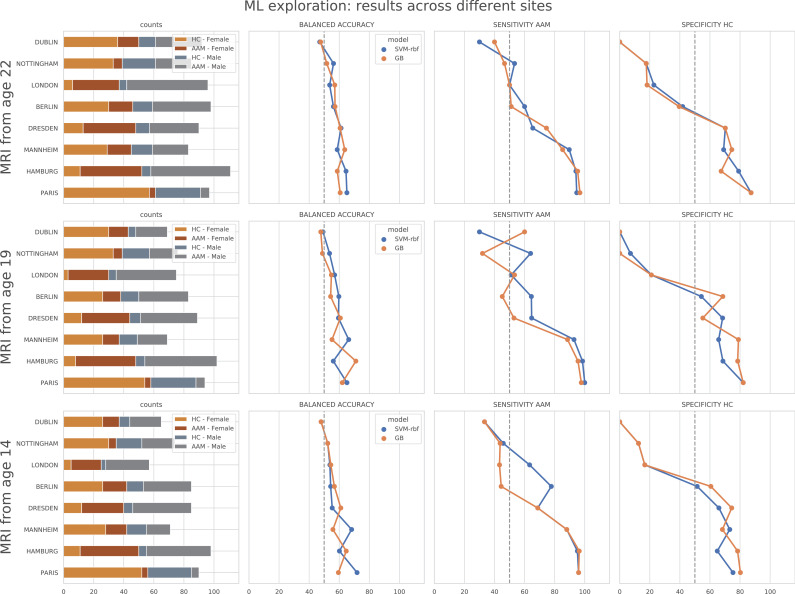
ML exploration results per site: Accuracy of the non-linear models per site in the main experiments. The sites are ordered from low to high accuracy.

**Appendix 1—figure 3.**
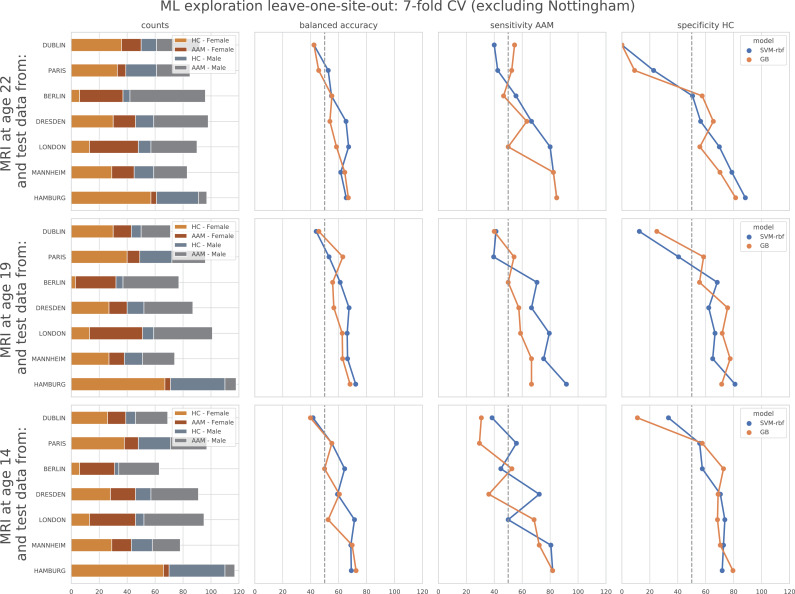
ML exploration results per site in *leave-one-site-out*: Accuracy of the non-linear models per site in the *leave-one-site-out*. The sites are ordered from low to high accuracy.

## Data Availability

This is a computational study. All data analyses code including the modelling pipeline are openly provided publicly at https://github.com/RoshanRane/ML_for_IMAGEN, (copy archived at swh:1:rev:6c493672ed700ded73c2b77e8976a5551921e634) for reuse and reproduction. Approval to use the IMAGEN dataset for this study was provided under the approval username / project code 'edeman'.

## Additional files

Transparent reporting form

## Appendix 1

**Appendix 1—table 1.** Hyperparameters: Each machine learning (ML) model has a set of hyperparameters that are tuned using an inner 5-fold cross-validation during the ML exploration stage. For both C and, γ higher values lead to overfitting and lower values can lead to underfitting. For gradient boosting, the maximum depth of the trees is set at, 5 the maximum numbers of estimators at, 100 and the subsampling of input features is disabled as counterbalancing is used. The remaining parameters are set at the default values as defined in the *scikit-learn* python package.

Model	hyperparameter	values tested
Logistic regression	C: Inverse of L2 regularization strength	1000, 100, 1.0, 0.001
Linear support vector machine	C: Inverse of L2 regularization strength	1000, 100, 1.0, 0.001
Kernel-basedsupport vector machine	C: Inverse of L2 regularization strengthγ: kernel coefficient of RBF kernel	1000, 100, 1.0, 0.001’auto’, ’scale’
Gradient boosting	learning_rate	0.05, 0.25

**Appendix 1—table 2.** AAM phenotype categorization: The table explains how the ten AAM phenotypes are derived from the respective IMAGEN questionnaire. It lists the total values in that question and what range of values are used to categorize the subjects into safe users, moderate users and heavy users, respectively. For reference, the sample sizes (n) obtained at FU3 by using these value ranges are also shown in the brackets.

Phenotype	IMAGEN questionnaire	Totalrange	Safe users *range (n*)	Moderate misusers*range (n*)	Heavy misusers *range (n*)
Frequency	ESPAD 8b	0-6	0-4 (397)	5 (270)	6 (372)
Amount	AUDIT q2	0-4	0 (413)	1 (403)	2-4 (219)
Onset	ESPAD 29d	11-21	16-21 (531)	14-15 (288)	11-14 (216)
Binge	ESPAD 19a	0-6	0-3 (299)	4-5 (336)	6 (400)
Binge-growth	Growth curveof ESPAD 19b	0-9	0-2 (379)	3-5 (420)	6-9 (236)
AUDIT	AUDIT-total	0-40	0-4 (443)	5-7 (274)	8-40 (318)
AUDIT-quick	AUDIT-freq	0-12	0-3 (402)	4-5 (359)	6-12 (274)
AUDIT-growth	Growth curveof AUDIT-total	0-6	0,3 (377)	4 (404)	2,5,6 (254)
Combined-seo	ESPAD 8b, 17b, 19b,and TLFB alcohol2	0-2	0 (345)	1 (404)	2 (286)
Combined-ours	AUDIT q1, q2,ESPAD 19a, growthcurve of ESPAD 19b	0-3	0 (429)	1 (403)	2 (203)

**Appendix 1—table 3.** Most informative sMRI features: An exhaustive list of the ‘most informative’ features in all three time point analyses provided along with their obtained SHAP values across seven repetitions. SHAP values that didn’t surpass the threshold are shown in italic. (Acronyms: area: surface area, volume: gray matter volume, thickness: average thickness, thicknessstd: standard deviation of thickness, intensity: mean intensity, meancurv: integrated rectified mean curvature, gauscurv: integrated rectified gaussian curvature, curvind: intrinsic curvature index, foldind: folding index;)

Feature					avg.	avg.	SHAP value						
Modality	Region	Side	Name	Type	Feature value	SHAP value	run1	run2	run3	run4	run5	run6	run7
FU3 (no. features = 21, threshold≥ 0.008743)													
DTI			Splenium of the corpus callosum		-0.87353	0.014433	0.014127	0.013667	0.015137	0.015569	0.016892	0.014402	0.0112
T1w	Cortical	Right	Lateral occipital cortex	*Thickness*	-0.677426	0.013378	0.012873	0.011353	0.012539	0.014382	0.014206	0.017304	0.011
T1w	Subcortical		Cerebrospinal fluid	*Intensity*	0.7903	0.013244	0.015039	0.013902	0.014382	0.014225	0.014667	0.009284	0.0112
T1w	Cortical	Left	Caudal anterior cingulate cortex	*Foldind*	-0.61388	0.012721	0.011392	0.012186	0.014931	0.014588	0.011412	0.017284	0.0073
T1w	Subcortical		Brain-Stem	*Intensity*	-0.59437	0.012637	0.011784	0.010304	0.013049	0.014657	0.017569	0.010069	0.011
T1w	Subcortical	Right	Amygdala	*Volume*	0.664147	0.012564	0.017804	0.01148	0.015137	0.012049	0.013353	0.008324	0.0098
T1w	Cortical	Right	Parahippocampal gyrus	*Area*	0.770722	0.012542	0.012373	0.010137	0.01449	0.015745	0.010049	0.015265	0.0097
T1w	Cortical	Right	Cuneus cortex	*Thickness*	-0.634456	0.012373	0.014275	0.012196	0.013461	0.01198	0.010049	0.012686	0.012
T1w	Subcortical	Right	Hippocampus	*Intensity*	0.623355	0.0122	0.015461	0.007725	0.010941	0.012794	0.013255	0.009765	0.0155
T1w	Subcortical	Left	Hippocampus	*Intensity*	0.663395	0.011909	0.014422	0.010049	0.011314	0.011843	0.011098	0.012098	0.0125
T1w	Subcortical	Left	Choroid-plexus	*Volume*	-0.761621	0.011884	0.010059	0.009392	0.015353	0.012922	0.013667	0.010049	0.0117
T1w	Cortical	Right	Rostral anterior cingulate cortex	*Thickness*	0.636769	0.011794	0.011853	0.009108	0.015412	0.014863	0.013775	0.009451	0.0081
T1w	Subcortical		Anterior corpus callosum	*Intensity*	-0.612797	0.01137	0.009333	0.010373	0.012843	0.006441	0.014314	0.017451	0.0088
T1w	Cortical	Left	Pericalcarine cortex	*Meancurv*	-0.724256	0.011364	0.012657	0.011157	0.014539	0.010422	0.01301	0.015637	0.0021
T1w	Cortical	Right	Superior parietal cortex	*Thickness*	-0.630512	0.011321	0.01051	0.010608	0.011216	0.012647	0.013245	0.013833	0.0072
T1w	Cortical	Right	Parahippocampal gyrus	*Meancurv*	-0.791755	0.010913	0.011686	0.013382	0.007471	0.011735	0.010373	0.010843	0.0109
DTI		Right	Retrolenticular part of the internal capsule		-0.712437	0.010793	0.009843	0.009667	0.012775	0.011961	0.010137	0.010343	0.0108
T1w	Cortical	Left	Lateral orbitofrontal cortex	*Meancurv*	0.696262	0.010761	0.011951	0.01098	0.011627	0.004167	0.011157	0.01548	0.01
T1w	Cortical	Left	Rostral anterior cingulate cortex	*Thickness*	-0.746005	0.010644	0.009824	0.01249	0.01049	0.010588	0.012235	0.012471	0.0064
T1w	Cortical	Right	Supramarginal gyrus	*Thickness*	-0.808923	0.010111	0.009627	0.005529	0.009333	0.009245	0.012451	0.013186	0.0114
DTI		Left	Hippocampal component of the cingulum		-0.727728	0.009451	0.010951	0.00901	0.009392	0.00902	0.011167	0.007373	0.0092
FU2 (no. features = 32, threshold≥ 0.009865)													
T1w	Cortical	Right	Caudal anterior cingulate cortex	*Curvind*	1.591756	0.019167	0.018951	0.018882	0.018324	0.018314	0.019235	0.021304	0.0192
T1w	Cortical	Left	Caudal anterior cingulate cortex	*Thicknessstd*	-0.746035	0.01701	0.01849	0.013353	0.022441	0.017098	0.015265	0.017804	0.0146
T1w	Cortical	Left	Cuneus cortex	*Curvind*	0.462589	0.016139	0.017657	0.015353	0.015284	0.014755	0.016118	0.018186	0.0156
T1w	Cortical	Right	Pars traingularis	*Thicknessstd*	-0.81719	0.015997	0.011755	0.013353	0.018539	0.023647	0.022029	0.007843	0.0148
T1w	Cortical	Left	Pericalcarine	*Curvind*	0.01016	0.015952	0.013755	0.017059	0.012667	0.017382	0.018863	0.015912	0.016
T1w	Cortical	Right	Inferior temporal gyrus	*Thicknessstd*	-0.854132	0.015746	0.014696	0.013696	0.023922	0.015373	0.019108	0.016951	0.0065
T1w	Subcortical		Anterior corpus callosum	*Intensity*	-0.642157	0.015396	0.018255	0.015137	0.015118	0.012314	0.016137	0.017559	0.0133
T1w	Cortical	Right	Cuneus cortex	*Thickness*	-0.72579	0.014955	0.010167	0.015471	0.018951	0.017706	0.015343	0.013118	0.0139
T1w	Cortical	Left	Pars opercularis	*Volume*	-0.628446	0.014697	0.016353	0.019078	0.018431	0.012941	0.01851	0.011588	0.006
DTI		Left	Corticospinal tract		0.775736	0.014336	0.013892	0.014314	0.015265	0.015912	0.014304	0.015549	0.0111
T1w	Subcortical		White matter	*Intensity*	-0.754712	0.01421	0.015765	0.015255	0.010118	0.015951	0.014853	0.015941	0.0116
T1w	Cortical	Right	Frontal pole	*Thickness*	0.619691	0.014148	0.017647	0.013255	0.013608	0.017275	0.012696	0.016333	0.0082
T1w	Cortical	Left	Pars opercularis	*Area*	-0.582627	0.014141	0.018157	0.01598	0.014745	0.013235	0.017412	0.011471	0.008
T1w	Cortical	Left	Frontal pole	*Curvind*	0.732718	0.014078	0.015412	0.016422	0.017882	0.015725	0.010412	0.010196	0.0125
T1w	Subcortical	Left	Cerebellum cortex	*Intensity*	0.610253	0.01399	0.012304	0.01652	0.016275	0.014392	0.014235	0.01251	0.0117
T1w	Cortical	Right	Precentral gyrus	*Gauscurv*	0.299008	0.013556	0.014127	0.011441	0.010725	0.013304	0.016206	0.01451	0.0146
T1w	Cortical	Left	Rostral anterior cingulate cortex	*Thickness*	-0.916001	0.013268	0.010324	0.013882	0.01348	0.01351	0.01249	0.015892	0.0133
T1w	Cortical	Left	Caudal anterior cingulate cortex	*Meancurv*	-0.704352	0.013246	0.010931	0.008147	0.020755	0.015137	0.014196	0.011843	0.0117
T1w	Subcortical		Brain-Stem	*Intensity*	-0.736592	0.013246	0.0105	0.015314	0.014471	0.014127	0.015059	0.013	0.0103
T1w	Cortical	Left	Fusiform gyrus	*Thicknessstd*	0.758297	0.013178	0.012922	0.009735	0.016853	0.01	0.015471	0.013049	0.0142
T1w	Cortical	Left	Lingual gyrus	*Thicknessstd*	0.725356	0.013094	0.011804	0.017461	0.006804	0.014157	0.01602	0.01299	0.0124
T1w	Cortical	Left	Pars opercularsis	*Meancurv*	-0.7511	0.013024	0.010667	0.01552	0.014235	0.013314	0.018245	0.013098	0.0061
T1w	Cortical	Left	Inferior temporal gyrus	*Thicknessstd*	0.73171	0.013018	0.010529	0.010167	0.014961	0.017627	0.011363	0.014961	0.0115
T1w	Cortical	Right	Banks of the superior temporal sulcus	*Meancurv*	-0.766809	0.012685	0.014314	0.011863	0.014951	0.012461	0.0145	0.013363	0.0073
T1w	Subcortical	Right	Accumbens area	*Intensity*	0.640973	0.01263	0.011108	0.012392	0.013255	0.014971	0.016147	0.011137	0.0094
T1w	Cortical	Right	Inferior parietal cortex	*Area*	0.844075	0.012417	0.015324	0.008647	0.011196	0.01401	0.011784	0.012951	0.013
T1w	Cortical	Left	Pericalcarine cortex	*Thickness*	-0.738264	0.012266	0.010647	0.011167	0.014951	0.01648	0.011618	0.011922	0.0091
T1w	Cortical	Right	Pars opercularis	*Area*	-0.562211	0.012139	0.010824	0.012941	0.013902	0.013304	0.015216	0.010029	0.0088
T1w	Subcortical	Left	Cerebellum white matter	*Intensity*	0.747148	0.012045	0.012363	0.01052	0.017598	0.015078	0.012284	0.011902	0.0046
T1w	Cortical	Left	Superior parietal cortex	*Thicknessstd*	0.725889	0.012003	0.011598	0.011794	0.011843	0.016029	0.010637	0.009363	0.0128
T1w	Cortical	Right	Postcentral gyrus	*Curvind*	0.84478	0.011399	0.008569	0.014353	0.012353	0.012971	0.010343	0.010392	0.0108
T1w	Subcortical	Left	Inferior lateral ventricle	*Volume*	-0.602808	0.010917	0.014912	0.009882	0.01049	0.011059	0.01051	0.009441	0.0101
BL (no. features = 46, threshold≥ 0.00993)													
T1w	Subcortical	Right	Pallidum	*Volume*	0.775721	0.023244	0.020892	0.019804	0.018647	0.022333	0.025186	0.030343	0.0255
T1w	Cortical	Left	Temporal pole	*Volume*	0.777293	0.021441	0.019167	0.018696	0.019637	0.02052	0.02452	0.023716	0.0238
T1w	Subcortical	Right	Cerebellum cortex	*Volume*	0.830438	0.020328	0.023157	0.023118	0.018931	0.017265	0.019608	0.022049	0.0182
T1w	Subcortical		Anterior corpus callosum	*Intensity*	-0.711844	0.01865	0.020873	0.021353	0.012127	0.019637	0.023373	0.012922	0.0203
T1w	Cortical	Left	Rostral middle frontal gyrus	*Thicknessstd*	-0.772379	0.018557	0.020049	0.016147	0.013402	0.02051	0.019088	0.019049	0.0217
T1w	Cortical	Right	Parahippocampal gyrus	*Area*	0.82387	0.018141	0.023725	0.012539	0.014431	0.021775	0.016961	0.017618	0.0199
T1w	Cortical	Right	Inferior parietal cortex	*Volume*	0.747201	0.018085	0.018373	0.018549	0.012686	0.020765	0.01851	0.019971	0.0177
T1w	Cortical	Left	Lateral occipital cortex	*Thickness*	-0.733224	0.017517	0.017627	0.014441	0.014373	0.025794	0.018529	0.018667	0.0132
T1w	Cortical	Right	Banks of the superior temporal sulcus	*Meancurv*	-0.7106	0.016707	0.019931	0.019559	0.015196	0.013127	0.01498	0.014706	0.0195
T1w	Cortical	Right	Parahippocampal gyrus	*Volume*	0.882348	0.016598	0.020588	0.013324	0.011706	0.020304	0.015363	0.017765	0.0171
T1w	Cortical	Left	Pericalcarine cortex	*Thickness*	-0.693217	0.015763	0.015657	0.010235	0.013569	0.019176	0.016049	0.022069	0.0136
DTI			Posterior corona radiata		-0.7244	0.015693	0.020441	0.015324	0.011775	0.018667	0.015873	0.015784	0.012
T1w	Cortical	Right	Superior parietal cortex	*Thicknessstd*	0.751469	0.015426	0.018333	0.017088	0.013176	0.020196	0.009814	0.016098	0.0133
DTI		Right	Posterior corona radiata		-0.697344	0.015406	0.020461	0.016431	0.011333	0.012902	0.019804	0.012245	0.0147
T1w	Cortical	Left	Paracentral lobule	*Area*	-0.695895	0.014994	0.012765	0.013294	0.013824	0.012775	0.01199	0.023284	0.017
T1w	Cortical	Left	Pars orbitalis	*Area*	0.756453	0.014944	0.015892	0.013441	0.011363	0.014167	0.014245	0.016922	0.0186
T1w	Cortical	Left	Superior parietal cortex	*Thicknessstd*	0.681101	0.014908	0.016196	0.01598	0.009784	0.012431	0.012775	0.022716	0.0145
T1w	Cortical	Right	Cuneus cortex	*Volume*	-0.753021	0.014805	0.015284	0.014608	0.010255	0.012039	0.016402	0.017392	0.0177
T1w	Cortical	Right	Pericalcarine cortex	*Thickness*	-0.599115	0.014742	0.016441	0.014245	0.013108	0.013588	0.016255	0.016108	0.0135
T1w	Cortical	Left	Rostral anterior cingulate cortex	*Curvind*	0.861164	0.014357	0.019578	0.017206	0.0125	0.013127	0.006725	0.011539	0.0198
T1w	Cortical	Right	Inferior parietal cortex	*Area*	0.778327	0.014151	0.016353	0.016559	0.007843	0.015961	0.015176	0.013078	0.0141
T1w	Cortical	Right	Cuneus cortex	*Thickness*	-0.650155	0.014062	0.01401	0.012755	0.014706	0.012755	0.013451	0.018059	0.0127
DTI		Right	Retrolenticular part of internal capsule		-0.715854	0.013992	0.011765	0.017833	0.00751	0.014333	0.016422	0.013725	0.0164
T1w	Cortical	Right	Inferior parietal cortex	*Thicknessstd*	0.710387	0.013828	0.014637	0.013706	0.014118	0.012402	0.008814	0.017324	0.0158
T1w	Subcortical		Anterior corpus callosum	*Volume*	0.828249	0.013824	0.014667	0.014951	0.014147	0.016588	0.012225	0.010637	0.0135
T1w	Cortical	Left	Medial orbitofrontal cortex	*Thicknessstd*	0.75355	0.013815	0.018461	0.013059	0.011608	0.009706	0.01999	0.013627	0.0103
T1w	Subcortical	Left	Cerebellum cortex	*Volume*	0.815826	0.013696	0.014559	0.012245	0.015069	0.008245	0.01551	0.017686	0.0126
T1w	Cortical	Right	Pars opercularis	*Thickness*	-0.752197	0.01369	0.015	0.015784	0.005745	0.01399	0.018029	0.013127	0.0142
DTI		Right	Anterior limb of internal capsule		0.776713	0.013662	0.010971	0.013343	0.013647	0.011853	0.015196	0.013559	0.0171
T1w	Cortical	Right	Transveretemporal cortex	*Thickness*	0.693202	0.013654	0.012941	0.014343	0.008676	0.018784	0.014314	0.01152	0.015
T1w	Cortical	Right	Isthmus cingulate cortex	*Thicknessstd*	0.654339	0.013653	0.014667	0.012931	0.011961	0.010461	0.019618	0.013275	0.0127
T1w	Cortical	Right	Medial orbitofrontal cortex	*Meancurv*	0.867091	0.013538	0.012412	0.010245	0.008775	0.018255	0.012078	0.01652	0.0165
DTI		Left	Posterior corona radiata		-0.69722	0.013132	0.015314	0.011696	0.010088	0.017137	0.01102	0.017343	0.0093
DTI		Left	Corticospinal tract		0.823927	0.013063	0.017108	0.014441	0.011216	0.013373	0.013186	0.012765	0.0094
T1w	Cortical	Left	Lateral orbitofrontal cortex	*Meancurv*	0.771492	0.012777	0.010775	0.015059	0.006745	0.018373	0.014176	0.01202	0.0123
T1w	Subcortical		Brain-Stem	*Intensity*	-0.791678	0.012619	0.015882	0.011539	0.010951	0.010461	0.011814	0.011667	0.016
DTI			Splenium of corpus callosum		-0.817257	0.012545	0.011627	0.010441	0.013176	0.014029	0.013196	0.015147	0.0102
T1w	Cortical	Left	Medial orbitofrontal cortex	*Area*	-0.768057	0.012541	0.014549	0.012225	0.011353	0.011039	0.011059	0.011706	0.0159
T1w	Cortical	Left	Paracentral lobule	*Volume*	-0.72805	0.012517	0.010078	0.010402	0.011431	0.012176	0.009196	0.020353	0.014
T1w	Subcortical	Left	Inferior lateral ventricle	*Volume*	-0.541217	0.01238	0.013245	0.010324	0.013863	0.013676	0.010186	0.009676	0.0157
T1w	Cortical	Right	Pericalcarine cortex	*Volume*	-0.62829	0.012329	0.011176	0.014784	0.010078	0.010451	0.011029	0.015794	0.013
T1w	Cortical	Left	Isthmus cingulate cortex	*Volume*	0.87903	0.012158	0.010794	0.011353	0.011186	0.011725	0.009412	0.013716	0.0169
T1w	Cortical	Left	Temporal pole	*Thicknessstd*	-0.780085	0.011993	0.011049	0.011735	0.012118	0.015598	0.007608	0.012618	0.0132
T1w	Cortical	Right	Isthmus cingulate cortex	*Meancurv*	0.859662	0.011721	0.012039	0.015029	0.01152	0.012657	0.010284	0.010451	0.0101
DTI			Retrorenticular part of internal capsule		-0.745934	0.010933	0.010765	0.010637	0.00401	0.013343	0.011833	0.014029	0.0119
DTI		Left	Inferior fronto-occipital fasciculus		0.914131	0.010721	0.010794	0.013069	0.005735	0.012745	0.010363	0.011108	0.0112
