# Supplementary material for: Structural differences in adolescent brains can predict alcohol misuse
Source: eLife. 2022 May 26;11:e77545. doi: 10.7554/eLife.77545 (PMC9255959; doi:10.7554/eLife.77545)
Supplement: Peer review history

# Editor report: Editor's evaluation

**DOI:** 10.7554/eLife.77545.sa0

**Authors:** Saad Jbabdi

**Affiliations:** https://ror.org/052gg0110 University of Oxford United Kingdom

This study uses a large dataset on alcohol misuse in adolescents that have been followed up for several years. MRI data are used to test whether the structure and connectivity of the brains of adolescents can predict their alcohol misuse later in their early twenties. The results show that binge drinking can be predicted out of multiple brain phenotypes with good accuracy, even after controlling for many confounding variables. This study can be impactful as it suggests a re-evaluation of studies of the effect of alcohol on the adolescent brain.

# Decision letter

**DOI:** 10.7554/eLife.77545.sa1

**Affiliations:** https://ror.org/052gg0110 University of Oxford United Kingdom

Our editorial process produces two outputs: (i) public reviews designed to be posted alongside the preprint for the benefit of readers; (ii) feedback on the manuscript for the authors, including requests for revisions, shown below. We also include an acceptance summary that explains what the editors found interesting or important about the work.

**Decision letter after peer review:**

Thank you for submitting your article "Structural differences in adolescent brains can predict alcohol misuse" for consideration by *eLife*. Your article has been reviewed by 2 peer reviewers, and the evaluation has been overseen by a Reviewing Editor and Chris Baker as the Senior Editor. The reviewers have opted to remain anonymous.

The reviewers have discussed their reviews with one another, and the Reviewing Editor has drafted this to help you prepare a revised submission.

Essential revisions:

Please pay particular attention to the below points raised by one of the reviewers, which I believe to be important.

1) Provide a more convincing explanation for the poor results of the leave-one-site-out predictions.

2) Model longitudinal changes using pre-onset data.

Reviewer #1 (Recommendations for the authors):

This study uses a nice longitudinal dataset and performs relatively thorough methodological comparisons. I also appreciate the systematic literature review presented in the introduction. The discussion of confound control is interesting and it is great that a leave-one-site-out test was included. However, the prediction accuracy drops in these important leave-one-site-out analyses, which should be assessed and discussed further. Furthermore, I think there is a missed opportunity to test longitudinal prediction using only pre-onset individuals to gain clearer causal insights. Please find specific comments below, approximately in order of importance.

1. The leave-one-site-out results fail to achieve significant prediction accuracy for any of the phenotypes. This reveals a lack of cross-site generalizability of all results in this work. The authors discuss that this variance could be caused by distributed sample sizes across sites resulting in uneven folds or site-specific variance. It should be possible to test these hypotheses by looking at the relative performance across CV folds. The site-specific variance hypothesis may be likely because for the other results confounds are addressed using oversampling (i.e., sampling with replacement) which creates a large sample with lower variance than a random sample of the same size. This is an important null finding that may have important implications, so I do not think that it is cause for rejection. However, it is a key element of this paper and I think it should be assessed further and discussed more widely in the abstract and conclusion.

2. The authors state that "83.3% of subjects reported having no or just one binge drinking experience until age 14". To gain clearer insights into the causality, I recommend repeating the MRIage14 → AAMage22 prediction using only these 83% of subjects.

3. The feature importance results for brain regions are quite inconsistent across time points. As such, the study doesn't really address one of the main challenges with previous work discussed in the introduction: "brain regions reported were not consistent between these studies either and do not tell a coherent story". This would be worth looking into further, for example by looking at other indices of feature importance such as permutation-based measures and/or investigating the stability of feature importance across bootstrapped CV folds.

*Reviewer #2 (Recommendations for the authors):*

I only have a few suggestions.

1) The abstract should be more specific, pointing out the best-predicted measure(binge) and the conclusion of methods, like the conclusion did. Also, the introduction should include more recent prediction studies using IMAGEN.

2) The structure of brain mapping needs clarification. We would like to see the most contributing structural measures（thickness, volume, intensity）separately mapping, how about the accuracy using each type alone? Could you show the brain mapping of each type of 3 sMRI features respectively? Figure 7 is too general.

3) How about the availability of data and code?

# Reply: Author response

**DOI:** 10.7554/eLife.77545.sa2

Reviewer #1 (Recommendations for the authors):

This study uses a nice longitudinal dataset and performs relatively thorough methodological comparisons. I also appreciate the systematic literature review presented in the introduction. The discussion of confound control is interesting and it is great that a leave-one-site-out test was included. However, the prediction accuracy drops in these important leave-one-site-out analyses, which should be assessed and discussed further. Furthermore, I think there is a missed opportunity to test longitudinal prediction using only pre-onset individuals to gain clearer causal insights. Please find specific comments below, approximately in order of importance.

We thank the reviewers for their positive remarks and for providing important suggestions to improve the analysis. Please see our detailed comments below.

1. The leave-one-site-out results fail to achieve significant prediction accuracy for any of the phenotypes. This reveals a lack of cross-site generalizability of all results in this work. The authors discuss that this variance could be caused by distributed sample sizes across sites resulting in uneven folds or site-specific variance. It should be possible to test these hypotheses by looking at the relative performance across CV folds. The site-specific variance hypothesis may be likely because for the other results confounds are addressed using oversampling (i.e., sampling with replacement) which creates a large sample with lower variance than a random sample of the same size. This is an important null finding that may have important implications, so I do not think that it is cause for rejection. However, it is a key element of this paper and I think it should be assessed further and discussed more widely in the abstract and conclusion.

We thank the reviewer for raising this point and providing specific suggestions. As mentioned by the reviewer, the *leave-one-site-out* results showed high-variance across sites, that is, across cross validation (CV) folds. Therefore, as suggested by the reviewer, we further investigated the source of this variance by observing how the model accuracies correlates with each site and its sample sizes, ratio of AAM-to-controls, and the sex distribution in each site. We ranked the sites from low to high accuracy and observed different performance metrics such as sensitivity and specificity:

As shown in Appendix—figure 3, the models performed close-to-chance for sites ‘Dublin’, ‘Paris’ and ‘Berlin’ (<60% mean balanced accuracy) in the *leave-one-site-out* experiment, across all time-points and metrics. Notably, the order of the performance at each site does not correspond to the sample sizes (please refer to the ‘counts’ column in the above figure). It also does not correspond to the ratio of AAM-to-controls, or to the sex distribution.

To further investigate this, we performed another additional *leave-one-site-out* experiment with all 8 sites. Here, we repeated the ML (Machine Learning) exploration by using the entire data, including the data from the Nottingham site that was kept aside as the holdout. Since there are 8 sites now, we used a 8-fold cross validation and observed how the model accuracy varied across each site:

**Author response image 1.** 

The results were comparable to the original leave-one-site-out experiment. Along with ‘Dublin’ and Berlin’, the models additionally performed poorly on the ‘Nottingham’ site. Results on ‘London’ and ‘Paris’ also fell below 60% mean balanced accuracy.Finally, we compared the above two results to the main experiment from the paper where the test samples were randomly sampled across all sites. The performance on test subjects from each site was compared:

As seen in Appendix—figure 2, the models struggled with subjects from ‘Dublin’ followed by ‘Nottingham’ ‘London’ and ‘Berlin’ respectively, and performed well on subjects from ‘Dresden’, ‘Mannheim’, ‘Hamburg’ and ‘Paris’.

Across all the three results discussed above, the models consistently struggle to generalize to subjects particularly from ‘Dublin’ and ‘Nottingham’. As already pointed out by the reviewer, the variance in the main experiment in the manuscript is lower because of the random sampling of the test set across all sites. Since these results have important implications, we have included them in the manuscript and also provided these figures in the Appendix.

2. The authors state that "83.3% of subjects reported having no or just one binge drinking experience until age 14". To gain clearer insights into the causality, I recommend repeating the MRIage14 → AAMage22 prediction using only these 83% of subjects.

3. The feature importance results for brain regions are quite inconsistent across time points. As such, the study doesn't really address one of the main challenges with previous work discussed in the introduction: "brain regions reported were not consistent between these studies either and do not tell a coherent story". This would be worth looking into further, for example by looking at other indices of feature importance such as permutation-based measures and/or investigating the stability of feature importance across bootstrapped CV folds.

Reviewer #2 (Recommendations for the authors):

I only have a few suggestions.

1) The abstract should be more specific, pointing out the best-predicted measure(binge) and the conclusion of methods, like the conclusion did. Also, the introduction should include more recent prediction studies using IMAGEN.

Thank you for the valuable feedback. We agree with these suggestions. We have now revised the abstract to include these important points and have also included more recent IMAGEN studies in the introduction, as shown below. Not all newly added studies are included in Table 1 because this table specifically enlists studies that look at AAM (adolescent alcohol misuse) and use structural MRI data.

Revised Abstract:

“Alcohol misuse during adolescence (AAM) has been associated with disruptive development of adolescent brains. In this longitudinal machine learning (ML) study, we could predict AAM significantly from brain structure (T1-weighted imaging and DTI) with accuracies of 73 – 78% in the IMAGEN dataset (n ~1182). Our results not only show that structural differences in brain can predict AAM, but also suggests that such differences might precede AAM behavior in the data. We predicted ten phenotypes of AAM at age 22 using brain MRI features at ages 14, 19, and 22. Binge drinking was found to be the most predictable phenotype. The most informative brain features were located in the ventricular CSF, and in white matter tracts of the corpus callosum, internal capsule, and brain stem. In the cortex, they were spread across the occipital, frontal, and temporal lobes and in the cingulate cortex. We also experimented with four different ML models and several confound control techniques. Support Vector Machine (SVM) with rbf kernel and Gradient Boosting consistently performed better than the linear models, linear SVM and Logistic Regression. Our study also demonstrates how the choice of the predicted phenotype, ML model, and confound correction technique are all crucial decisions in an explorative ML study analyzing psychiatric disorders with small effect sizes such as AAM.”

New IMAGEN citations:

35. Irina Filippi, Andre´ Galinowski, Herve´ Lemaˆıtre, Christian Massot, Pascal Zille, Pauline Fre`re, Rube´n Miranda-Marcos, Christian Trichard, Stella Guldner, He´le`ne Vulser, et al. Neuroimaging evidence for structural correlates in adolescents resilient to polysubstance use: A five-year follow-up study. European Neuropsychopharmacology, 49:11–22, 2021.

36. Tianye Jia, Chao Xie, Tobias Banaschewski, Gareth J Barker, Arun LW Bokde, Christian Bu¨chel, Erin Burke Quinlan, Sylvane Desrivie`res, Herta Flor, Antoine Grigis, et al. Neural network involving medial orbitofrontal cortex and dorsal periaqueductal gray regulation in human alcohol abuse. Science Advances, 7(6):eabd4074, 2021.

37. Sarah Yip, Fengdan Ye, Qinghao Liang, Sarah Lichenstein, Alecia Dagher, Godfrey Pearlson, Bader Charani, Hugh Garavan, and Dustin Scheinost. Neuromarkers of risky alcohol use from age 14 to 19 years. Biological Psychiatry, 91(9):S41–S42, 2022.

2) The structure of brain mapping needs clarification. We would like to see the most contributing structural measures（thickness, volume, intensity）separately mapping, how about the accuracy using each type alone? Could you show the brain mapping of each type of 3 sMRI features respectively? Figure 7 is too general.

The contributions of different structural feature types was analysed initially during the *ML exploration* stage, using the 'Binge growth' phenotype. Author response image 2 shows the results from this analysis. Using a single feature type such as only thickness, only volume, or only area resulted in an accuracy that is 6-9% lower than when all features were used together. It suggests that when all features are provided together, the ML models can additionally exploit certain mutual (multivariate) information, that might be present across these different feature types. Therefore, we decided to use all features as input rather than individual feature types and then observe the feature importance post-hoc.

**Author response image 2.** 

Nevertheless, we have now provided a table consisting of all the features and feature types with their respective SHAP scores across seven repetitions in the Appendix. This table is aimed to enable future researchers to further investigate the relative contributions of different features and feature types.

3) How about the availability of data and code?

This information has now been provided explicitly in the Data Availability section.
